# Effect of *-NBOMe* Compounds on Sensorimotor, Motor, and Prepulse Inhibition Responses in Mice in Comparison With the *2C* Analogs and Lysergic Acid Diethylamide: From Preclinical Evidence to Forensic Implication in Driving Under the Influence of Drugs

**DOI:** 10.3389/fpsyt.2022.875722

**Published:** 2022-04-21

**Authors:** Micaela Tirri, Sabrine Bilel, Raffaella Arfè, Giorgia Corli, Beatrice Marchetti, Tatiana Bernardi, Federica Boccuto, Giovanni Serpelloni, Francesco Botrè, Fabio De-Giorgio, Krystyna Golembiowska, Matteo Marti

**Affiliations:** ^1^Section of Legal Medicine and Laboratory for Advanced Therapy Technologies (LTTA) Centre, Department of Translational Medicine, University of Ferrara, Ferrara, Italy; ^2^Department of Chemistry and Pharmaceutical Sciences, University of Ferrara, Ferrara, Italy; ^3^Neuroscience Clinical Center and Transcranial Magnetic Stimulation (TMS) Unit, Verona, Italy; ^4^Institute of Sport Science University of Lausanne (ISSUL), Lausanne, Switzerland; ^5^Section of Legal Medicine, Department of Health Care Surveillance and Bioethics, Università Cattolica del Sacro Cuore, Rome, Italy; ^6^Fondazione Policlinico Universitario A. Gemelli Istituto di Ricovero e Cura a Carattere Scientifico (IRCCS), Rome, Italy; ^7^Department of Pharmacology, Maj Institute of Pharmacology, Polish Academy of Sciences, Krakòw, Poland; ^8^Department of Anti-Drug Policies, Collaborative Center for the Italian National Early Warning System, Presidency of the Council of Ministers, Rome, Italy

**Keywords:** LSD, phenethylamine, DUID (driving under the influence of drugs), novel psychoactive substances (NPS), pre-pulse inhibition, sensorimotor, 2C compounds, -NBOMe

## Abstract

In the last decade, the market for new psychoactive substances has been enriched by numerous psychedelic phenethylamines, which mimic the psychoactive effect of lysergic acid diethylamide (LSD). In particular, the -NBOMe series, which are more potent than their 2C compounds analogs, are considered worthy substitutes for LSD by users. The purpose of this study was to assess the effects of 25*H*-NBOMe and its halogenated derivatives (25*I*-NBOMe and 25*B*-NBOMe) in comparison to their 2C compounds analogs and LSD on the sensorimotor (visual, acoustic, and overall tactile), reaction time, spontaneous (total distance traveled) and stimulated (drag, accelerod test) motor activity, grip strength test, and prepulse inhibition (PPI) responses in mice. Systemic administration of -NBOMe, 2C compounds analogs, and LSD (0.001–10 mg/kg) differently impaired the sensorimotor, reaction time, motor, and PPI responses in mice. In particular, halogenated (25I and 25B)-NBOMe derivatives appear to be more effective than the entire class of 2C compounds analogs in altering visual and acoustic responses, affecting reaction time, and motor and sensory gating in PPI test. In fact, the specific rank order of compounds potency for nearly all of the experiments showed that (25I and 25B)-NBOMe were more potent than 2C compounds analogs and LSD. -NBOMe and 2C compounds analogs impaired not only the reception of incoming sensory stimuli (visual and acoustic), but their correct brain processing (PPI) in an equal and sometimes stronger way than LSD. This sensory impairment directly affected the spontaneous motor response and reaction time of mice, with no change in performance in stimulated motor activity tests. These aspects should be carefully considered to better understand the potential danger that psychedelic phenethylamines, in particular -NBOMe, may pose to public health, with particular reference to decreased performance in driving and hazardous works that require special sensorimotor skills.

## Introduction

The worldwide increase in number and type of new psychoactive substances (NPSs) seems to be a potential risk factor for public health (1, 2). Commonly, illicit use of NPSs through different mechanisms of action leads to direct or indirect activation of catecholaminergic and serotonergic mechanisms that progressively result in clinical outcomes (3, 4). For example, hallucinogens have become increasingly popular among NPSs with serotoninergic action (5) and have also been found among the so-called “club-drugs,” substances used by young adults (15–34 years old) who prefer traditional oral administration (tablets or blotters), insufflation or smoking (6) to induce changes in perception and cognitive states (7, 8).

Pharmacologically, the psychedelic effects of hallucinogenic compounds are mainly mediated by their ability to activate, as full or partial agonists, the 5HT_2A_ serotonergic receptor (7, 9–12), since genetic or pharmacological inactivation of the 5-HT_2A_ receptor blocks the behavioral effects of hallucinogenic compounds in both mice and rats (13–15). However, experiments performed on animal models, also in humans, proved that the psychedelic effect of hallucinogenic compounds may also be due to their agonism on other serotonin receptors, such as 5-HT_2C_ (16) and 5-HT_1A_ (17).

**FIGURE 1:**
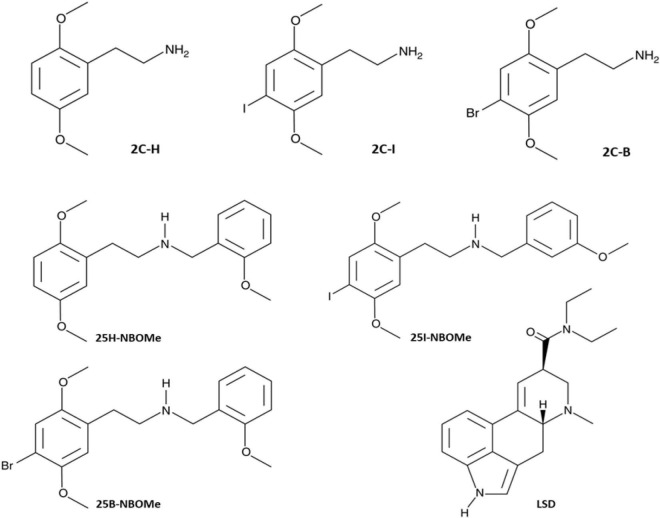
Chemical structures of 2,5-dimethoxyphenethylamine (2C-H), 2,5-Dimethoxy-4-iodophenethylamine (2C-I), 2,5-dimethoxy-4-bromophenethylamine (2C-B), 2,5-dimethoxy-N-[(2-methoxyphenyl) methyl]-benzeneethanamine (25H-NBOMe), 4-iodio-2,5-dimethoxy-N-[(2-methoxyphenyl) methyl]-benzeneethanamine (25I-NBOMe), 4-bromo-2,5-dimethoxy-N-[(2-methoxyphenyl) methyl]-benzeneethanamine (25B-NBOMe), and Lysergic acid diethylamide (LSD).

Nowadays, psychedelic serotonergic drugs are classified into three main groups based on their chemical structure: tryptamines (including psilocin and its analogs), lysergamides [including lysergic acid diethylamide (LSD) and its analogs], and phenethylamines (including mescalin and its analogs) (18). Interestingly, (+)-LSD (Figure 1) has been the most popular hallucinogen since the mid-1960s and has been found to have high affinity for most serotonin receptors (11). Therefore, LSD is considered a very potent serotoninergic hallucinogen, and it is also becoming a valid research tool for comparing the effects of much more widespread NPSs or “research chemicals” (19, 20) belonging to the same class. In fact, among the best-selling substances in the illicit drug market as feasible LSD replacement molecules are -NBOMe and its parent compounds, the 2C compounds series. All molecules that belong to these two series (-NBOMe and 2C compounds series), even if chemically different from LSD, produce intense psychedelic and dysperceptive effects (21). Previous *in vivo* studies have confirmed that 25B-NBOMe shares hallucinogenic activity with other classical hallucinogens, such as 2,5-Dimethoxy-4-iodoamphetamine [DOI; (22, 23)], LSD and mescaline (23). Despite continuous national and international monitoring systems of the illicit drug market, phenethylamines are so sought among NPSs that the 2C-B is among the top 10 in several European countries (1) and also the most prevalent psychedelic NPS used in the United States (24). Subsequently, the advent of -NBOMe series with extremely powerful activities toward the 5-HT_2A_ receptor and strong hallucinogenic effects, exceeded the use of the popular 2C compounds (6, 21, 25–27). However, Erowid’s research highlights a large number of experience narratives from both phenethylamine series (28). Among the short-term dose-dependent effects reported by users is the ability of -NBOMe compounds to alter the mind; state of consciousness; perception of time and space; individual emotional range; self-perception; and a profound alteration in visual, auditory, tactile, and olfactory perception. However, the same users also report side effects, such as increased blood pressure, heart rate, and body temperature; dizziness, loss of appetite and dry mouth; increased sweating, decreased impulsivity and rapid emotional change (from fear to euphoria in a matter of seconds); numbness, weakness, and tremors. In contrast, the toxicological syndrome caused by the ingestion of -NBOMe compounds is characterized by evident neuropsychiatric effects (agitation, delirium, perceptive disorders, and seizures) and autonomous instabilities (tachycardia, hypertension, diaphoresis, and pupil dilation) ranging from medium to severe (21, 29–31).

In the last 10 years, many preclinical studies have focused on assessing the hallucinogenic and psychedelic effects of phenethylamines. Preclinical *in vitro* studies clearly demonstrated the affinity of LSD and several 2C compounds analogs for serotonergic receptors, showing in particular that -NBOMe series are ultrapotent and highly efficacious agonists of serotonin 5-HT_2A_ and 5-HT_2C_ receptors (Ki values in low nanomolar range) with more than 1000-fold selectivity for 5-HT_2A_ compared with 5-HT_1A_. The -NBOMe compounds display higher affinity for 5-HT_2A_ receptors than their 2C counterparts and have markedly lower affinity, potency, and efficacy for the 5-HT_2B_ receptor than the 5-HT_2A_ or 5-HT_2C_ (21, 26, 32, 33).

In contrast, *in vivo* studies have become most popular for pharmacological investigation of behavioral proxy in rodents to determine human hallucinogenic effects. In fact, there are certain tests that can reliably distinguish between hallucinogenic and non-hallucinogenic 5-HT_2A_ receptor agonists, such as head twitch response, prepulse inhibition (PPI), and behavioral pattern monitor in rodents (16, 20, 34, 35). Many studies focused on the possible alteration of the auditory impulse processing capacities following the administration of hallucinogenic substances in rodents, and their response was used as a factor for the recognition of hallucinogenic psychoactive power on animals’ sensory gating (36–38). However, the relevance of the hallucinatory state and the sensory impairment caused by -NBOMe, 2C compounds, and LSD in modifying motor reactivity and behavior in mice has not been investigated.

This aspect from a translational point of view is of considerable importance to underline the danger of these compounds, not only for their acute toxicity on the body, but to highlight their danger in cases of intake in people driving or who must perform complex motor tasks.

Therefore, we investigated and compared the effects of 25*H*-NBOMe and its halogenated derivatives (25*I*-NBOMe and 25*B*-NBOMe) with their 2C compounds analogs and LSD on the sensorimotor (visual, acoustic, and overall tactile) responses; reaction time; spontaneous motor activity expressed as total distance traveled and stationing in C zone; stimulated motor activity expressed as drag, accelerod, and grip strength test; and startle PPI test in mice.

## Materials and Methods

### Animals

Adult male ICR (CD-1^®^) mice weighing 30–35 g (Centralized Preclinical Research Laboratory, University of Ferrara, Italy) were group-housed (5 mice per cage, 80 cm^2^ floor area per animal, and 12 cm minimum enclosure height), exposed to a 12:12 h light-dark cycle (light period from 6:30 a.m. to 6:30 p.m.) at a temperature of 20–22°C and humidity of 45–55%, and provided *ad libitum* access to food (Diet 4RF25 GLP; Mucedola, Settimo Milanese, Milan, Italy) and water. The experimental protocols performed in the present study were in accordance with the U.K. Animals (Scientific Procedures) Act of 1986 and its associated guidelines and the new European Communities Council Directive of September 2010 (2010/63/EU). Experimental protocols were approved by the Italian Ministry of Health (license no. 335/2016-PR) and by the Animal Welfare Body of the University of Ferrara. According to the ARRIVE guidelines, all possible efforts were made to minimize the number of animals used, minimize the animals’ pain and discomfort, and reduce the number of experimental subjects. In safety pharmacology studies (visual, acoustic, and tactile sensorimotor responses; reaction time test; muscle strength; and drag and accelerod test) for 2,5-dimethoxy-N-[(2-methoxyphenyl) methyl]-benzeneethanamine (25H-NBOMe), 4-iodio-2,5-dimethoxy-N-[(2-methoxyphenyl) methyl]-benzeneethanamine (25I-NBOMe), 4-bromo-2,5-dimethoxy-N-[(2-methoxyphenyl) methyl]-benzeneethanamine (25B-NBOMe), 2,5-dimethoxyphenethylamine (2C-H), 2,5-dimethoxy-4-iodophenethylamine (2C-I), 2,5-dimethoxy-4-bromophenethylamine (2C-B), and Lysergic acid diethylamide (LSD) experiments, eight mice (total mice used: 336) were used for each treatment (vehicle or 5 doses; 0.001, 0.01, 0.1, 1, and 10 mg/kg). Similarly, in the analysis of spontaneous locomotion (open field test) for 25H-NBOMe, 25I-NBOMe, 25B-NBOMe, 2C-H, 2C-I, 2C-B, and LSD experiments, eight mice (total mice used: 336) were used for each treatment (vehicle or 5 doses; 0.001, 0.01, 0.1, 1, and 10 mg/kg). Lastly, in the PPI test for 25H-NBOMe, 25I-NBOMe, 25B-NBOMe, 2C-H, 2C-I, 2C-B, and LSD experiments, eight mice (total mice used: 272) were used for each treatment (vehicle or 5 different doses0.001 mg/kg, 0.01, 0.1, 1, and 10 mg/kg for –NBOMe compounds; and vehicle or 3 different doses: 0.1, 1, and 10 mg/kg for 2C compounds and LSD).

### Drug Preparation and Animal Dose Determination

25H-NBOMe, 25I-NBOMe, 25B-NBOMe, 2C-H, 2C-I, 2C-B, and LSD were purchased from LGC Standards S.r.L. (Milan, Italy). Drugs were dissolved in Tween 80 (2%) and ethanol (5%), brought to the final volume with saline (0.9% NaCl), and administered by intraperitoneal (i.p.) injection at a volume of 4 μL/gr. Tween 80 (2%), ethanol (5%), and saline were also used as the vehicle.

Doses of 25H-NBOMe, 25I-NBOMe, 25B-NBOMe, 2C-H, 2C-I, 2C-B, and LSD were chosen based on the behavioral and neurological effects reported on internet experiences (39), different intake rates among users, and duration of effect [Tables 1, 2, respectively; (40)]. Therefore, the lowest dose tested in this study (0.001 mg/kg) is equivalent to a human sub-threshold dose. The intermediate dosages tested range between threshold/common dosages for human-*NBOMe* and light/high dosages for human LSD, while the higher dosages tested (1 and 10 mg/kg) exceed the highest values consumed by users (40). In addition, previous *in vivo* studies have already confirmed the effectiveness of -*NBOMe* and 2C compounds doses chosen in this study to test mice (31, 34, 41).

**TABLE 1:** Different dosages of hallucinogenic compounds (40).

Dosage	Hallucinogenic compounds						
	25H-NBOMe	25I-NBOMe	25B-NBOMe	2C-H	2C-I	2C-B	LSD
Threshold	/	50 μg	50 μg	/	2 mg	5 mg	15 μg
Light	/	200–500 μg	100–300 μg	/	5–10 mg	10–15 mg	25–75 μg
Common	/	500–700 μg	300–500 μg	/	10–20 mg	15–25 mg	75–150 μg
Strong	/	700–1,000 μg	500–700 μg	/	20–30 mg	25–45 mg	150–300 μg
Heavy	/	/	/	/	>30 mg	>45 mg	>300 μg

**TABLE 2:** Different duration of effects of hallucinogen compounds (40).

Duration of effects	Hallucinogenic compounds						
	25H-NBOMe	25I-NBOMe	25B-NBOMe	2C-H	2C-I	2C-B	LSD
Total	/	360–600 min	480–720 min	/	360–600 min	300–480 min	480–720 min
Onset	/	15–120 min	20–40 min	/	15–45 min	20–40 min	13–20 min
Come up	/	30–120 min	30–90 min	/	45–90 min	40–60 min	45–90 min
Peak	/	120–240 min	240–360 min	/	180–300 min	120–180 min	180–300 min
Offset	/	60–240 min	120–240 min	/	120–180 min	90–180 min	180–300 min
After effects	/	3–6 days	120–360 min	/	360–1,440 min	120–240 min	720–2,880 min

### Behavioral Tests

The effects of 25H-NBOMe, 25I-NBOMe, 25B-NBOMe, 2C-H, 2C-I, 2C-B, and LSD were investigated using a consolidated battery of behavioral tests routinely used in our laboratory for preclinical investigation of NPSs in rodents (38, 42, 43).

To reduce the number of animals used, mice were evaluated using different tests carried out in a consecutive manner according to the following time scheme. Observation of visual object responses (frontal and lateral view), acoustic response and tactile response (pinna, vibrissae, and corneal reflexes), were measured 10, 30, 60, 120, 180, 240, and 300 min after injection of the compounds, while visual placing response was measured at 15, 35, 65, 125, 185, 245, and 305 min. Reaction time and accelerod tests were measured at 15, 40, 70, 130, 190, 250, and 310 min, while drag test and muscle strength were measured at 45, 70, 105, 160, 220, 280, and 340 min. For the visual object, acoustic, and tactile sensorimotor tests, each mouse was housed in an experimental chamber (350 × 350 × 350 mm) made with black methacrylate walls and a transparent front door. A camera (B/W USB Camera day and night with varifocal lens; Ugo Basile, Italy) was placed at the top and/or side of the box. To avoid mice olfactory cues, cages were carefully cleaned with a dilute (5%) ethanol solution and washed with water.

Effects of 25H-NBOMe, 25I-NBOMe, 25B-NBOMe, 2C-H, 2C-I, 2C-B, and LSD on spontaneous locomotion were investigated using the ANY-maze video-tracking system (application version 4.99 g Beta; Stoelting Co., Europe, Dublin, Ireland), while for alteration of acoustic sensorimotor gating, the startle/PPI technique (Ugo Basile apparatus, Milan, Italy) was used.

Behavioral tests were conducted in the Centralized Preclinical Research Laboratory of University of Ferrara at a thermostated temperature of 20–22°C, humidity of approximately 45–55%, and controlled light (approximately 150 lx) with a background noise of approximately 40 ± 4 dB.

All experiments were performed from 8:30 a.m. to 2:00 p.m. Experiments were conducted in blind by trained observers working together in pairs (44).

#### Evaluation of the Visual Response

The visual response was verified using two behavioral tests which evaluated the ability of the animal to capture visual information when the animal was either stationary (the visual object response) or moving (the visual placing response) (for technical details of the methods used, see Supplementary Material).

#### Evaluation of Acoustic Response

Acoustic response measures the reflex of the mouse in response to an acoustic stimulus (four acoustic stimuli of different intensities and frequencies were tested) produced behind the animal (for technical details of the methods used, see Supplementary Material).

#### Evaluation of Tactile Response

Tactile response of the mouse was verified through vibrissae, corneal, and pinnae reflexes. Data are expressed as the sum of the three afore-mentioned parameters (for technical details of the methods used, see Supplementary Material).

#### Evaluation of Reaction Time

This test was performed to measure the animal’s motor reactivity in the open field (45). Mice were accustomed by placing them in the center of a square area (150 × 150 cm) for 5 min, then lifted from the tail to 3 cm above the surface and finally dropped. When the animal touched the floor, the latency time for the first movement of the front limb was recorded. Usually, the average reaction time of the mouse is about 0.21 s (for technical details of the methods used, see Supplementary Material).

#### Evaluation of Skeletal Muscle Strength

This test was used to evaluate the skeletal muscle strength of the mice (45). The grip-strength apparatus (ZP-50N, IMADA) comprises of a wire grid (5 × 5 cm) connected to an isometric force transducer (dynamometer). In the grip-strength test, mice were held by their tails and allowed to grasp the grid with their forepaws (for technical details of the methods used, see Supplementary Material).

#### Stimulated Motor Activity Assessment

Alterations of stimulated motor activity induced by 25H-NBOMe, 25I-NBOMe, 25B-NBOMe, 2C-H, 2C-I, 2C-B, and LSD were measured using two tests in which the animal is forced to move (drag test and accelerod). In the drag test the mouse was lifted by the tail, leaving the front paws on the table and dragged backward at a constant speed of about 20 cm/s for a fixed distance (100 cm). The number of steps taken by each paw was recorded by two different observers. For each animal, five to seven measurements were performed. For the accelerod test, animals were placed at 5-min intervals on a rotating cylinder whose speed automatically and constantly increased (0–60 rotations/min). Time spent on the cylinder was then measured, placing a cut-off at 300 s (for technical details of the methods used, see Supplementary Material).

#### Spontaneous Locomotor Activity

Spontaneous locomotor activity (distance traveled and time in the central C1 zone) was measured using the ANY-maze video tracking system (Ugo Basile, application version 4.99 g Beta). In the open field test, 25H-NBOMe, 25I-NBOMe, 25B-NBOMe, 2C-H, 2C-I, 2C-B, and LSD were administered intraperitoneally (i.p.) in mice and each animal was singly placed in the open field box (for technical details of the methods used, see Supplementary Material).

#### Startle/Prepulse Inhibition Test

Mice were tested for acoustic startle reactivity and PPI responses in startle chambers (Ugo Basile apparatus, Milan, Italy) consisting of a sound-attenuated, lighted, and ventilated enclosure holding a transparent non-restrictive Perspex^®^ cage (90 × 45 × 50 mm). All acoustic stimuli were produced through a loudspeaker mounted lateral to the holder. A load cell was then able to detect the peak and startle response amplitudes. At the onset of the startling stimulus, 300-ms readings were recorded and the wave amplitude evoked by the movement of the animal’s startle response was measured (for technical details of the methods used, see Supplementary Material).

### Data and Statistical Analysis

In sensorimotor response experiments, data are expressed in arbitrary units (visual object response, acoustic response, overall tactile response) and percentages of baseline (visual placing response, reaction time, time on rod, number of steps, and muscle strength). To calculate total sensory inhibition, the values expressed in basal units (object and acoustic) were converted into percentages. Data from spontaneous locomotion studies are expressed in absolute values for the total distance traveled (m) and the time spent in the central C1 zone (seconds). To calculate the inhibition of the total distance traveled, the indicated absolute values (expressed in meters) have been converted into a percentage of the baseline. The amount of PPI was calculated as a percentage score for each prepulse + pulse trial type:% PPI = 100 − {[(startle response for prepulse + pulse trial)/(startle response for pulse-alone trial)] × 100}. Startle magnitude was calculated as the average response to all the pulse-alone trials. All numerical data are given as mean ± SEM of four independent experimental replications. Statistical analysis of the effects of the individual substances in different concentrations over time was performed by two-way ANOVA followed by Bonferroni’s test for multiple comparisons, while statistical analysis of PPI results was carried out with one-way ANOVA followed by the Bonferroni’s test for multiple comparisons and expressed in histograms. Statistical analysis of the correlation between the sensory dysperception with reaction time and total distance traveled by mice was performed by *t*-test.

All statistical analysis were performed with the Prism software (GraphPad Prism, United States).

## Results

### Behavioral Studies

#### Sensorimotor Studies

##### Evaluation of the Visual Object Response

**FIGURE 2:**
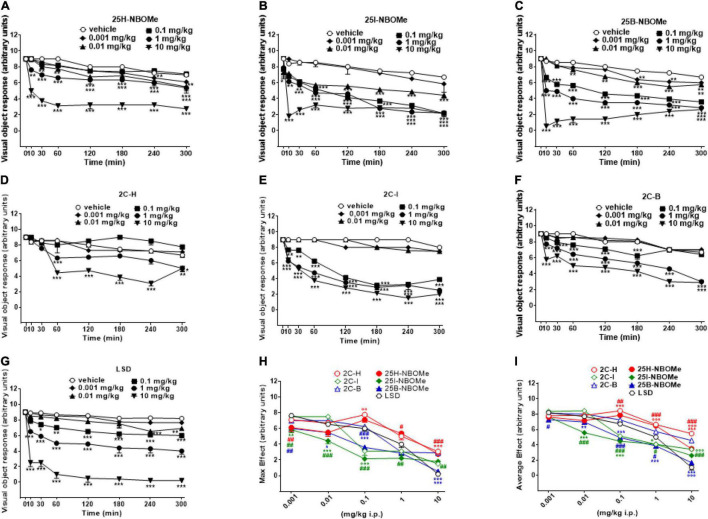
Effect of 25H-NBOMe (0.001–10 mg/kg i.p.) **(A)** 25I-NBOMe (0.001–10 mg/kg i.p.) **(B)** 25B-NBOMe (0.001–10 mg/kg i.p.) **(C)** 2C-H (0.001–10 mg/kg i.p.) **(D)** 2C-I (0.001–10 mg/kg i.p.) **(E)** 2C-B (0.001–10 mg/kg i.p.) **(F),** and LSD (0.001–10 mg/kg i.p.) **(G)** on the visual object tests in mice, and comparison of the maximum **(H)** and average **(I)** effects observed in 5 h. Data are expressed as mean ± SEM (*n* = 8/group). Statistical analysis was performed by two-way ANOVA followed by Bonferroni’s test **(A–G)** for multiple comparisons for the dose–response curve of each compound at different time points. The comparison of maximum effect observed in 5 h was also presented **(H,I)**. *p < 0.05, **p < 0.01, ***p < 0.001 vs. vehicle; ^#^*p* < 0.05, ^##^*p* < 0.01, ^###^*p* < 0.001 vs. LSD.

Visual object response was not affected by the administration of the vehicle over the course of the 5-h analysis (Figures 2A–G). However, the systemic administration of 25H-NBOMe (0.001–10 mg/kg i.p.) produced changes in visual object response (Figure 2A); two-way ANOVA revealed a major effect of treatment [*F*_(5,336)_ = 526.7, *p* < 0.0001], effect of time after injection [*F*_(7,336)_ = 207.8, *p* < 0.0001], and a significant time × treatment interaction [*F*_(35,336)_ = 13.46, *p* < 0.0001]. Similarly, 25I-NBOMe (0.001–10 mg/kg i.p.) affected the visual object response (Figure 2B); two-way ANOVA revealed a major effect of treatment [*F*_(5,336)_ = 357.1, *p* < 0.0001], effect of time after injection [*F*_(7,336)_ = 150.4, *p* < 0.0001], and a significant time × treatment interaction [*F*_(35,336)_ = 12.20, *p* < 0.0001]. The 25B-NBOMe (0.01–10 mg/kg i.p.) also inhibited the visual object response (Figure 2C); two-way ANOVA revealed a major effect of treatment [*F*_(5,336)_ = 2,334, *p* < 0.0001], effect of time after injection [*F*_(7,336)_ = 812.7, *p* < 0.0001], and a significant time × treatment interaction [*F*_(35,336)_ = 80.56, *p* < 0.0001]. Data have shown that 25H-NBOMe effects are less potent than its halogenated derivatives. Systemic administration of 2C-H (0.001–10 mg/kg i.p.) inhibited visual object response in mice (Figure 2D); two-way ANOVA revealed a major effect of treatment [*F*_(5,384)_ = 90.36, *p* < 0.0001], effect of time after injection [*F*_(7,384)_ = 55.13, *p* < 0.0001], and a significant time × treatment interaction [*F*_(35,384)_ = 9.923, *p* < 0.0001]. The 2C-I compound (0.001–10 mg/kg i.p.) also inhibited visual object response in mice (Figure 2E); two-way ANOVA revealed a major effect of treatment [*F*_(5,336)_ = 962.6, *p* < 0.0001], effect of time after injection [*F*_(7,336)_ = 293.9, *p* < 0.0001] and a significant time × treatment interaction [*F*_(35,336)_ = 34.73, *p* < 0.0001]. The 2C-B (0.001–10 mg/kg i.p.) affected visual object response in mice (Figure 2F); two-way ANOVA revealed a major effect of treatment [*F*_(5,336)_ = 1,051, *p* < 0.0001], effect of time after injection [*F*_(7,336)_ = 629.3, *p* < 0.0001], and a significant time × treatment interaction [*F*_(35,336)_ = 36.70, *p* < 0.0001]. Data show that the effects of 2C-H, similar to the hydrogenated molecules of the -NBOMe, are less potent in respect to its halogenated derivatives. In fact, 2C-H was effective at higher doses of 1 and 10 mg/kg, while 2C-I and 2C-B compounds inhibited visual object response at 0.1 mg/kg dose. Moreover, visual inhibition induced by 2C-I and 2C-B already appeared 10 min after their systemic administration, while that caused by 2C-H was delayed and significant at 60 min after the drug injection. It also seems that 2C-I effect was stronger than that of 2C-B.

Systemic administration of LSD (0.001–10 mg/kg i.p.) was effective in inhibiting visual object response in mice starting from a dose of 0.1 mg/kg (Figure 2G); two-way ANOVA revealed a major effect of treatment [*F*_(5,336)_ = 603.6, *p* < 0.0001], effect of time after injection [*F*_(7,336)_ = 102.5, *p* < 0.0001], and a significant time × treatment interaction [*F*_(35,336)_ = 13.45, *p* < 0.0001].

Thus, -NBOMe, 2C compounds, and LSD inhibit visual object response in mice in a dose-dependent manner; effects of -NBOMe and LSD are faster and stronger than those of 2C compounds.

The maximum effect of visual object response decrease induced by -NBOMe, 2C compounds, and *LSD* (Figure 2H) showed statistically significant results at all doses [*F*_(6,251)_ = 45.13, *p* < 0.0001]. The average effect presented in Figure 2I showed a different power and effectiveness of -NBOMe, 2C compounds, and LSD compounds [*F*_(6,251)_ = 96.71, *p* < 0.0001]. Furthermore, from the comparison of the maximum effects, it may be suggested that 2C compounds are less potent than -NBOMe and LSD. Based on the average effect analysis, the potency rating of the compounds may be presented as follows: 25I-NBOMe (ED_50_ = 0.1302 mg/kg) > 25B-NBOMe (ED_50_ = 0.2432 mg/kg) > 2C-I (ED_50_ = 0.5754 mg/kg) > LSD (ED_50_ = 1.046 mg/kg) > 25H-NBOMe (ED_50_ = 4.470 mg/kg) > 2C-B (ED_50_ = 6.307 mg/kg) > 2C-H (ED_50_ = 13.84 mg/kg).

##### Evaluation of the Visual Placing Response

**FIGURE 3:**
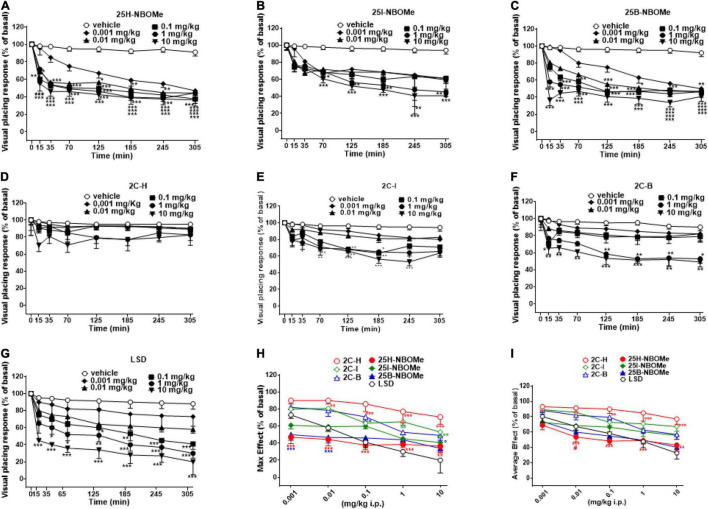
Effect of 25H-NBOMe (0.001–10 mg/kg i.p.) **(A)** 25I-NBOMe (0.001–10 mg/kg i.p.) **(B)** 25B-NBOMe (0.001–10 mg/kg i.p.) **(C)** 2C-H (0.001–10 mg/kg i.p.) **(D)** 2C-I (0.001–10 mg/kg i.p.) **(E)** 2C-B (0.001–10 mg/kg i.p.) **(F)** and LSD (0.001–10 mg/kg i.p.) **(G)** on the visual placing tests in the mice and comparison of the maximum **(H)** and average **(I)** effect observed in 5 h. Data are expressed as mean ± SEM (*n* = 8/group). Statistical analysis was performed by two-way ANOVA followed by Bonferroni’s test **(A–G)** for multiple comparisons for the dose–response curve of each compound at different time points. The comparison of maximum effect observed in 5 h was also presented **(H,I)**. *p < 0.05, **p < 0.01, ***p < 0.001 vs. vehicle; ^#^*p* < 0.05 vs. LSD.

Visual placing response tended to be unvaried in vehicle-treated mice over the 5-h observation period (Figures 3A–G). Systemic administration of 25H-NBOMe (0.001–10 mg/kg i.p.) reduced in a dose-dependent manner the visual placing response in mice and persisted for up to 5 h at higher doses (Figure 3A); two-way ANOVA revealed a major effect of treatment [*F*_(5,336)_ = 90.82, *p* < 0.0001], effect of time after injection [*F*_(7,336)_ = 64.08, *p* < 0.0001], and a significant time × treatment interaction [*F*_(35,336)_ = 2.900, *p* < 0.0001]. The visual placing response reduction was also induced by systemic administration of 25I-NBOMe (0.001–10 mg/kg i.p.; Figure 3B); two-way ANOVA revealed a significant effect of treatment [*F*_(5,336)_ = 35.18, *p* < 0.0001], effect of time [*F*_(7,336)_ = 29.17, *p* < 0.0001], and time × treatment interaction [*F*_(35,336)_ = 1.527, *p* = 0.0324]. Similarly, administration of 25B-NBOMe (0.001–10 mg/kg i.p.) reduced in a dose dependent manner the visual placing response in mice, which persisted for up to 5 h (Figure 3C); two-way ANOVA revealed a significant effect of treatment [*F*_(5,336)_ = 113.7, *p* < 0.0001], effect of time after injection [*F*_(7,336)_ = 67.73, *p* < 0.0001], and a significant time × treatment interaction [*F*_(35,336)_ = 4.614, *p* < 0.0001]. This data shows that the 25H-NBOMe seems to have a more immediate and stronger effect than its halogenated derivatives. Systemic administration of 2C-H (0.001–10 mg/kg i.p.) did not affect the visual placing response of mice (Figure 3D); two-way ANOVA revealed effect of treatment [*F*_(5,336)_ = 4.802, *p* = 0.0003], not significant effect of time [*F*_(7,336)_ = 1.767, *p* = 0.0930], and time × treatment interaction [*F*_(35,336)_ = 0.2911, *p* > 0.9999]. On the other hand, the treatment with halogenated compounds 2C-I (0.001–10 mg/kg i.p.) decreased the visual placing response in mice in a dose-dependent manner (Figure 3E); two-way ANOVA revealed a significant effect of treatment [*F*_(5,336)_ = 50.8, *p* < 0.0001], effect of time [*F*_(7,336)_ = 29.55, *p* < 0.0001], and time × treatment interaction [*F*_(35,336)_ = 1.806, *p* = 0.0045]. Similarly, 2C-B compound (0.001–10 mg/kg i.p.) also decreased visual placing response in mice in a dose-dependent manner (Figure 3F); two-way ANOVA revealed a significant effect of treatment [*F*_(5,336)_ = 40.96, *p* < 0.0001], effect of time [*F*_(7,336)_ = 15.75, *p* < 0.0001], and not significant time × treatment interaction [*F*_(35,336)_ = 1.404, *p* = 0.0696]. This data shows that 2C-I was more effective than 2C-B, as it inhibited visual object response at the dose of 0.1 mg/kg, and 2C-B at the dose of 1 mg/kg.

Systemic administration of LSD (0.001–10 mg/kg i.p.) inhibited the visual placing response in mice in a dose-dependent manner (Figure 3G); two-way ANOVA revealed a significant effect of treatment [*F*_(5,336)_ = 61.29, *p* < 0.0001], effect of time after injection [*F*_(7,336)_ = 30.47, *p* < 0.0001], and a significant time × treatment interaction [*F*_(35,336)_ = 1.556, *p* = 0.0266]. This data shows that the effect of LSD was more potent than that of 2C compounds and -NBOMe.

The maximum effects of decrease in visual placing response induced by -NBOMe, 2C compounds, and LSD clearly showed the dose-dependence effect of all substances [Figure 3H; effect of treatment *F*_(6,245)_ = 31.52, *p* < 0.0001)]. The analysis of the maximum inhibitory effects on the visual placing response showed that 2C compounds were less potent than -NBOMe and LSD. The average effect of -NBOMe, 2C compounds, and LSD on the visual placing response (Figure 3I) showed a significant effect of treatment [*F*_(6,245)_ = 24.7, *p* < 0.0001] and a different power and effectiveness of the compounds, which may be presented in ascending order as follows: 25H-NBOMe (ED_50_ = 0.09809 mg/kg) > LSD (ED_50_ = 0.5976 mg/kg) > 25B-NBOMe (ED_50_ = 0.7067 mg/kg) > 25I-NBOMe (ED_50_ = 7.144 mg/kg) > 2C-B (ED_50_ = 8.450 mg/kg) > 2C-I (ED_50_ = 15.66 mg/kg) > 2C-H (ED_50_ = 29.90 mg/kg).

##### Evaluation of the Acoustic Response

**FIGURE 4:**
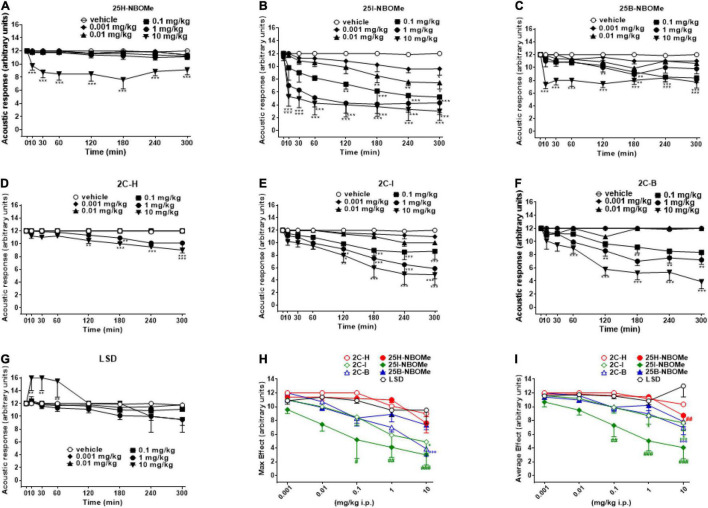
Effect of 25H-NBOMe (0.001–10 mg/kg i.p.) **(A)** 25I-NBOMe (0.001–10 mg/kg i.p.) **(B)** 25B-NBOMe (0.001–10 mg/kg i.p.) **(C)** 2C-H (0.001–10 mg/kg i.p.) **(D)** 2C-I (0.001–10 mg/kg i.p.) **(E)** 2C-B (0.001–10 mg/kg i.p.) **(F)**, and LSD (0.001–10 mg/kg i.p.) **(G)** on the acoustic tests in mice and comparison of the maximum **(H)** and average **(I)** effects observed in 5 h. Data are expressed as mean ± SEM (*n* = 8/group). Statistical analysis was performed by two-way ANOVA followed by Bonferroni’s test **(A–G)** for multiple comparisons for the dose–response curve of each compound at different time points. The comparison of maximum effect observed in 5 h was also presented **(H,I)**. *p < 0.05, **p < 0.01, ***p < 0.001 vs. vehicle; ^#^*p* < 0.05, ^##^*p* < 0.01, ^###^*p* < 0.001 vs. LSD.

The acoustic responses did not change in vehicle-treated mice over the 5-h observation period (Figures 4A–G). The treatment with 25H-NBOMe (0.001–10 mg/kg i.p.) decreased the acoustic responses in mice, only at higher doses (Figure 4A); two-way ANOVA revealed a significant effect of treatment [*F*_(5,336)_ = 53.33, *p* < 0.0001], effect of time [*F*_(7,336)_ = 3.629, *p* = 0.0009], and time × treatment interaction [*F*_(35,336)_ = 1.540, *p* = 0.0297]. Acoustic responses in mice were also decreased by 25B-NBOMe (0.001–10 mg/kg i.p.) (Figure 4C); two-way ANOVA revealed a significant effect of treatment [*F*_(5,336)_ = 50.50, *p* < 0.0001], effect of time [*F*_(7,336)_ = 11.53, *p* < 0.0001] and time × treatment interaction [*F*_(35,336)_ = 2.103, *p* = 0.0004]. Administration of 25I-NBOMe (0.001–10 mg/kg i.p.) reduced in a dose dependent manner the acoustic response in mice and the effect persisted for up to 5 h at higher doses (Figure 4B); two-way ANOVA revealed a significant effect of treatment [*F*_(5,336)_ = 45.87, *p* < 0.0001], effect of time [*F*_(7,336)_ = 12.25, *p* < 0.0001], and not significant time × treatment interaction [*F*_(35,336)_ = 1.171, *p* = 0.2388]. Administration of 2C-H (0.001–10 mg/kg i.p.) inhibited acoustic responses in mice, only at higher doses starting from 120 min after the injection (Figure 4D); two-way ANOVA revealed a significant effect of treatment [*F*_(5,336)_ = 61.09, *p* < 0.0001], effect of time [*F*_(7,336)_ = 11.43, *p* < 0.0001], and time × treatment interaction [*F*_(35,336)_ = 4.614, *p* < 0.0001]. The 2C-I (0.001–10 mg/kg i.p.) decreased acoustic responses in mice at higher doses (Figure 4E); two-way ANOVA revealed a significant effect of treatment [*F*_(5,336)_ = 31.60, *p* < 0.0001], effect of time [*F*_(7,336)_ = 16.52, *p* < 0.0001], and time × treatment interaction [*F*_(35,336)_ = 1.932, *p* = 0.0017]. The 2C-B (0.001–10 mg/kg i.p.) also reduced the acoustic response in mice at higher doses (Figure 4F). Two-way ANOVA revealed a significant effect of treatment [*F*_(5,344)_ = 80.60, *p* < 0.0001], effect of time after injection [*F*_(7,344)_ = 19.91, *p* < 0.0001], and a significant time × treatment interaction [*F*_(35,344)_ = 5.313, *p* < 0.0001]. Comparing the inhibitory acoustic effect induced by the 2C compounds, the dose-dependence, but the late manifestation (∼120 min after administration), was observed. The two halogenated compounds (2C-I and 2C-B) were more potent in their effect than 2C-H. However, the acoustic responses were not changed in LSD-treated mice at doses 0.001–10 mg/kg (Figure 4G). Two-way ANOVA revealed significant effect of treatment [*F*_(5,336)_ = 5.083, *p* = 0.0002], effect of time after injection [*F*_(7,336)_ = 5.890, *p* < 0.0001], and a significant time × treatment interaction [*F*_(35,336)_ = 1.665, *p* = 0.0126]. Contrary to the inhibitory profile of other substances, LSD seems to overexcite mice in the first hour of the test following administration. When comparing the effects of all compounds, -NBOMe and 2C compounds but not LSD were inhibitory in this test. The maximum effect in the acoustic response indicated that 25I-NBOMe was the strongest compound among the substances tested (Figure 4H), but the significant results are shown only for higher doses [0.1–10 mg/kg; *F*_(6,246)_ = 16.94, *p* < 0.0001]. The average effects on acoustic response showed significant results only for higher doses (0.1–10 mg/kg) of -NBOMe, 2C compounds, and LSD [Figure 4I; *F*_(6,246)_ = 19.84, *p* < 0.0001]. The power and effectiveness of the compounds may be shown in ascending order as follows: 25I-NBOMe (ED_50_ = 0.4585 mg/kg) > 2C-B (ED_50_ = 11.26 mg/kg) > 2C-I (ED_50_ = 13.82 mg/kg) > 25B-NBOMe (ED_50_ = 16.82 mg/kg) > 25H-NBOMe (ED_50_ = 26.64 mg/kg) > 2C-H (ED_50_ = 58.02 mg/kg). The power and effectiveness of LSD were not detected.

##### Evaluation of the Tactile Response

Overall tactile response did not change in vehicle groups and other groups of mice after administration of all substances studied in mice over the 5-h observation period (Supplementary Figures 1A–G). However, LSD (0.001–10 mg/kg i.p.) showed a dose-dependent effect of hyperstimulation on the tactile response (Figure 1G). Two-way ANOVA revealed a significant effect of treatment [*F*_(5,336)_ = 17.34, *p* < 0.0001], effect of time [*F*_(7,336)_ = 9.938, *p* < 0.0001], and not significant time × treatment interaction [*F*_(35,336)_ = 0.9953, *p* = 0.4803]. Similar effects can be seen in the acoustic response (Figure 4G).

##### Evaluation of Reaction Time

**FIGURE 5:**
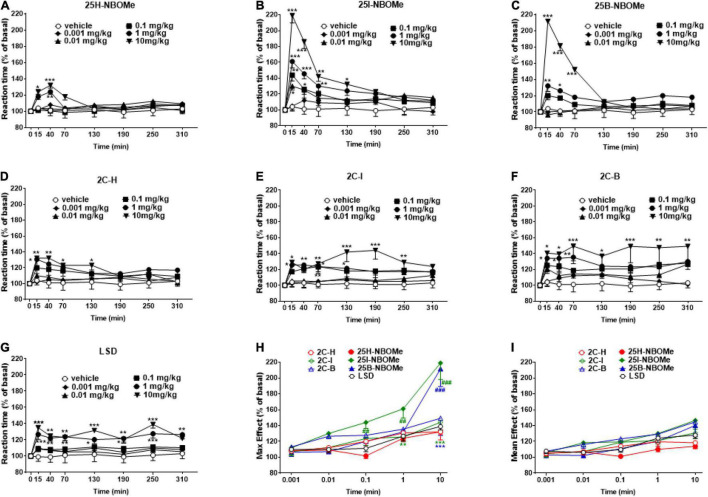
Effect of 25H-NBOMe (0.001–10 mg/kg i.p.) **(A)** 25I-NBOMe (0.001–10 mg/kg i.p.) **(B)** 25B-NBOMe (0.001–10 mg/kg i.p.) **(C)** 2C-H (0.001–10 mg/kg i.p.) **(D)** 2C-I (0.001–10 mg/kg i.p.) **(E)** 2C-B (0.001–10 mg/kg i.p.) **(F)**, and LSD (0.001–10 mg/kg i.p.) **(G)** on reaction time test in the mice and comparison of the maximum **(H)** and average **(I)** effect observed in 5 h. Data are expressed as mean ± SEM (*n* = 8/group). Statistical analysis was performed by two-way ANOVA followed by Bonferroni’s test **(A–G)** for multiple comparisons for the dose–response curve of each compound at different time points. The comparison of maximum effect observed in 5 h was also presented **(H,I)**. *p < 0.05, **p < 0.01, ***p < 0.001 vs. vehicle; ^##^*p* < 0.01, ^###^*p* < 0.001 vs. LSD.

The reaction time in vehicle-treated mice did not change over the 5-h observation period (Figures 5A–G). The treatment with 25H-NBOMe (0.001–10 mg/kg i.p.) slightly increased the reaction time of mice only at higher doses (1–10 mg/kg) for the first 40 min (Figure 5A). Two-way ANOVA revealed a significant effect of treatment [*F*_(5,302)_ = 6.452, *p* < 0.0001], effect of time [*F*_(7,302)_ = 3.571, *p* = 0.0010], and not significant time × treatment interaction [*F*_(35,302)_ = 1.382, *p* = 0.0809]. On the other hand, systemic administration of 25I-NBOMe (0.001–10 mg/kg i.p.) increased the reaction time of the animals, starting at 15 min after administration (Figure 5B); two-way ANOVA revealed a significant effect of treatment [*F*_(5,302)_ = 34.59, *p* < 0.0001], effect of time [*F*_(7,302)_ = 30.17, *p* < 0.0001], and time × treatment interaction [*F*_(35,302)_ = 4.522, *p* < 0.0001]. A similar effect was observed for 25B-NBOMe (0.001–10 mg/kg i.p.; Figure 5C); two-way ANOVA revealed a significant effect of treatment [*F*_(5,302)_ = 43.48, *p* < 0.0001], effect of time [*F*_(7,302)_ = 19.81, *p* < 0.0010], and time × treatment interaction [*F*_(35,302)_ = 8.211, *p* < 0.0001]. Thus, halogenated but not hydrogenated compounds significantly increased the reaction time of mice. Systemic administration of 2C-H (0.001–10 mg/kg i.p.) increased the response time of mice only during the first 2 h after injection (Figure 5D); two-way ANOVA revealed a significant effect of treatment [*F*_(5,302)_ = 11.94, *p* < 0.0001], effect of time [*F*_(7,302)_ = 4.911, *p* < 0.0001], and not significant time × treatment interaction [*F*_(35,302)_ = 0.9875, *p* = 0.4931]. The treatment with 2C-I (0.001–10 mg/kg i.p.) increased the reaction time of mice only at higher doses (0.1–10 mg/kg; Figure 5E); two-way ANOVA revealed a significant effect of treatment [*F*_(5_,_302)_ = 25.02, *p* < 0.0001], effect of time [*F*_(7,302)_ = 4.910, *p* < 0.0001], and not significant time × treatment interaction [*F*_(35,302)_ = 0.9856, *p* = 0.4961]. Systemic administration of 2C-B (0.001–10 mg/kg i.p.) increased the reaction time response in mice only at the highest dose (10 mg/kg), which persisted for up to 5 h (Figure 5F); two-way ANOVA revealed a significant effect of treatment [*F*_(5,302)_ = 20.09, *p* < 0.0001], effect of time after injection [*F*_(7,302)_ = 4.383, *p* = 0.0001], and not significant time × treatment interaction [*F*_(35,302)_ = 0.6422, *p* = 0.9434]. The comparison of effects of the 2C compounds clearly shows that halogenated compounds were stronger in increasing the reaction time of mice than 2C-H. However, the reaction time responses following LSD (0.001–10 mg/kg i.p.) increased only at higher doses (0.1–10 mg/kg) and the effect persisted for up to 5 h (Figure 5G); two-way ANOVA revealed a major effect of treatment [*F*_(5,302)_ = 28.91, *p* < 0.0001], effect of time [*F*_(7,302)_ = 5.536, *p* < 0.0010], and time × treatment interaction [*F*_(35,302)_ = 0.7891, *p* < 0.0001]. The maximum effects on the reaction time response increase induced by -NBOMe, 2C compounds, and LSD showed statistically significant results [*F*_(6,217)_ = 17.54, *p* < 0.0001] only at higher doses (0.1–10 mg/kg; Figure 5H). However, the average effects on reaction time response induced by -NBOMe, 2C compounds, and LSD did not show statistically significant results (Figure 5I). In fact, the power and effectiveness of different substances were not detected.

##### Correlation Between Sensory Dysperception and Reaction Time

**FIGURE 6:**
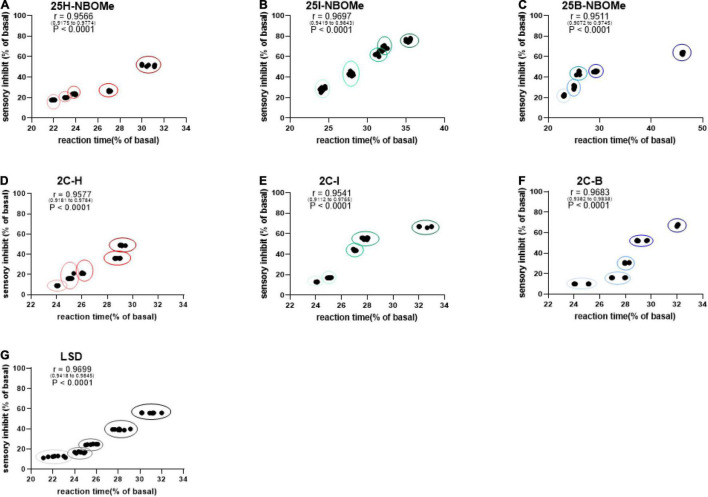
Correlation between sensory dysperception and reaction time of mice following administration of 25H-NBOMe (0.001–10 mg/kg i.p.) **(A)**, 25I-NBOMe (0.001–10 mg/kg i.p.) **(B)**, 25B-NBOMe (0.001–10 mg/kg i.p.) **(C)**, 2C-H. (0001–10 mg/kg i.p.) **(D)**, 2C-I (0.001–10 mg/kg i.p.) **(E)**, 2C-B (0.001–10 mg/kg i.p.) **(F)**, and LSD (0.001–10 mg/kg i.p.) **(G)** observed in 1 h. Data are expressed as mean ± SEM (*n* = 8/group). Statistical analysis was performed by XY correlation, which revealed a correlation between the two different effects of treatment. *p* < 0.0001.

To investigate whether the increase in reaction time during the first hour of administration in mice is proportional to their sensory inhibition, we considered evaluating the correlation between these parameters. Administration of the three -NBOMe compounds clearly showed different effects of correlation. For 25H-NBOMe (0.001–10 mg/kg i.p.; Figure 6A), XY correlation test revealed a correlation “r” between the two different effects of treatment [*r*_(0_._9175–0_._9774)_ = 0.9566, *p* < 0.0001]. The correlation profile for 25I-NBOMe (0.001–10 mg/kg i.p.) was similar to that of 2C-I (Figure 6B). However, XY correlation test for 25I-NBOMe (0.001–10 mg/kg i.p.; Figure 6B) revealed a correlation “r” between the two parameters [*r*_(0_._9419–0_._9843)_ = 0.9697, *p* < 0.0001]. It showed a higher sensory inhibition profile than for the other substances tested; however, a corresponding increase in reaction time of mice was similar to that seen in the 2C compounds. In contrast, the XY correlation test for 25B-NBOMe (0.001–10 mg/kg i.p.; Figure 6C) revealed a correlation “r” between the two different effects of treatment [*r*_(0_._9072–0_._9745)_ = 0.9511, *p* < 0.0001]. The increase in reaction time was proportional to sensory inhibition only at the highest dose tested (10 mg/kg).

The administration of the three 2C compounds clearly showed a different effect of correlation: XY correlation test for 2C-H (0.001–10 mg/kg i.p.; Figure 6D) revealed a correlation “r” between the two different effects of treatment [*r*_(0_._9181–0_._9784)_ = 0.9577, *p* < 0.0001], which shows a dose-dependent sensory inhibition with the peak of approximately 60% at the highest dose tested, and the reaction time increases proportionately in a dose-dependent manner (from approximately 24–29%). Differently, the presence of halogen shows a proportional increase in both values: for 2C-I (0.001–10 mg/kg i.p.; Figure 6E) the XY correlation test revealed a correlation “r” between the two different effects of treatment [*r*_(0_._9112–0_._9765)_ = 0.9541, *p* < 0.0001] and for 2C-B (0.001–10 mg/kg i.p.; Figure 6F), the XY correlation test revealed a correlation “r” between the two different effects of treatment [*r*_(0_._9382–0_._9838)_ = 0.9683, *p* < 0.0001]. The correlation test showed a normal (or a 0.26-s higher than normal) reaction time for sensory inhibition values of less than or equal to 20%, especially at the lowest dosages tested (0.001–0.1 mg/kg). At higher doses (1–10 mg/kg), sensory inhibition and reaction time of the mouse increased proportionately in a dose-dependent manner (from approximately 24 to 35% for 2C-I, and from approximately 22 to 46% for 2C-B).

Finally, the XY correlation test results obtained from the administration of LSD (0.001–10 mg/kg i.p.; Figure 6G) revealed a correlation “r” between the two different effects of treatment [*r*_(0_._9418–0_._9845)_ = 0.9699, *p* < 0.0001] and clearly showed a sharp dose-dependent correlation between sensory inhibition (max peak at 60% for highest dose tested) and increase in reaction time of mice (from 22 to 29% approximately).

##### Muscle Strength

The muscle strength test response did not change in both the vehicle and treatment substance administration in mice over the 5-h observation period (Supplementary Figures 2A–G).

##### Accelerod Test

The accelerod test response did not change in both the vehicle and treatment substance administration in mice over the 5-h observation period (Supplementary Figures 3A–G).

##### Drag Test

The drag test response did not change in both the vehicle and treatment substance administration in mice over the 5-h observation period (Supplementary Figures 4A–G).

##### Studies on Spontaneous Locomotor Activity in Mice

**FIGURE 7:**
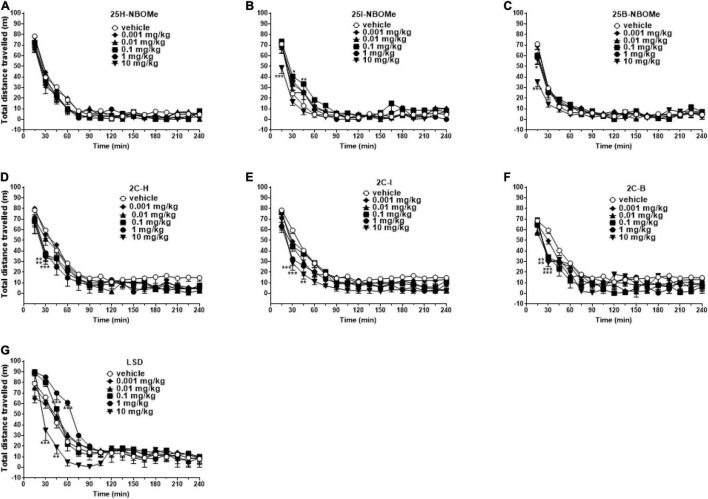
Effect of 25H-NBOMe (0.001–10 mg/kg i.p.) **(A)**, 25I-NBOMe (0.001–10 mg/kg i.p.) **(B)**, 25B-NBOMe (0.001–10 mg/kg i.p.) **(C)**, 2C-H (0.001–10 mg/kg i.p.) **(D)**, 2C-I (0.001–10 mg/kg i.p.) **(E)**, 2C-B (0.001–10 mg/kg i.p.) **(F)**, and LSD (0.001–10 mg/kg i.p.) **(G)** on the total distance traveled of mice over a 4-h observation period. Data are expressed as meters traveled and represent the mean ± SEM of eight determinations for each treatment. Statistical analysis was performed by two-way ANOVA followed by Bonferroni’s test for multiple comparisons for the dose–response curve of each compound at different times. *p < 0.05, **p < 0.01, ***p < 0.001 vs. vehicle.

**FIGURE 8:**
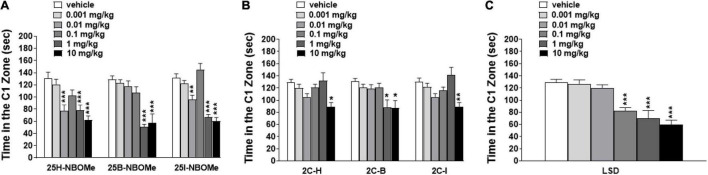
Effect of 25H-NBOMe (0.001–10 mg/kg i.p.), 25I-NBOMe (0.001–10 mg/kg i.p.), and 25B-NBOMe (0.001–10 mg/kg i.p.) **(A)**; 2C-H (0.001–10 mg/kg i.p.), 2C-I (0.001–10 mg/kg i.p.) and 2C-B (0.001–10 mg/kg i.p.) **(B)**; and LSD (0.001–10 mg/kg i.p.) **(C)** on the time spent in the C1 zone of mice during the 4-h observation period. Data are expressed as seconds and represent the mean ± SEM of 8 determinations for each treatment. Statistical analysis was performed by one-way ANOVA followed by Bonferroni’s test for multiple comparisons for the dose–response curve of each compound at different times. *p < 0.05, **p < 0.01, ***p < 0.001 vs. vehicle.

To exclude that the reduction in induced sensorimotor responses could be due to the inhibition of motor activity, we investigated the effect of -NBOMe, 2C compounds, and LSD administration (0.001–10 mg/kg i.p.) on spontaneous locomotor activity in mice (Figures 7A–G). The 25H-NBOMe (Figure 7A) is the only substance of the NBOMe series that does not show differences from the vehicle group. Conversely, 25I-NBOMe [Figure 7B; significant effect of treatment [*F*_(5,672)_ = 9.061, *p* < 0.0001], effect of time [*F*_(15,672)_ = 105.1, *p* < 0.0001], and time × treatment interaction [*F*_(75,672)_ = 1.086, *p* = 0.2976] and 25B-NBOMe [Figure 7C; significant effect of treatment [*F*_(4_,_560)_ = 4.918, *p* = 0.0055], effect of time [*F*_(15,560)_ = 105.2, *p* < 0.0001], and time × treatment interaction [*F*_(60,560)_ = 1.503, *p* = 0.0055] administration showed a statistically potent decrease in the total distance traveled only at a high dose (10 mg/kg) 15 min after injection. The 2C compounds did not show differences from the vehicle administration, except for the first hour of treatment: 2C-H [Figure 7D; significant effect of treatment [*F*_(5,672)_ = 9.940, *p* < 0.0001], effect of time [*F*_(15,672)_ = 93.57, *p* < 0.0001], and not significant time × treatment interaction [*F*_(75,672)_ = 0.6324, *p* = 0.9930], 2C-I [Figure 7E; significant effect of treatment [*F*_(5,672)_ = 19.71, *p* < 0.0001], effect of time [*F*_(15,672)_ = 97.12, *p* < 0.0001], and not significant time × treatment interaction [*F*_(75,672)_ = 0.5603, *p* = 0.9989], and 2C-B [Figure 7F; significant effect of treatment [*F*_(5,672)_ = 8.897, *p* < 0.0001], effect of time [*F*_(15,672)_ = 56.89, *p* < 0.0001], and not significant time × treatment interaction [*F*_(75,672)_ = 0.8259, *p* = 0.8499]. However, systemic administration of LSD [Figure 7G; significant effect of treatment [*F*_(5,672)_ = 11.02, *p* < 0.0001], effect of time [*F*_(15,672)_ = 102.5, *p* < 0.0001], and time × treatment interaction [*F*_(75,672)_ = 2.303, *p* < 0.0001] showed a stimulation of the total distance traveled for low doses (0.01–1 mg/kg) and an inhibition for the highest dose (10 mg/kg). In conclusion, a comparison of the effects of -NBOMe, 2C compounds, and LSD on the total distance traveled by mice shows an inhibitory effect only at the highest dose tested for all substances (10 mg/kg). In addition, low doses of LSD present stimulating effects. Subsequently, we analyzed the time spent in the center of the box during the first hour of registration (Figures 8A–C). LSD and -NBOMe decreased the time spent in the C zone at doses of 0.01–10 mg/kg. One-way ANOVA showed a significant effect of treatment for 25H-NBOMe [*F*_(5,42)_ = 9.467, *p* < 0.0001], 25I-NBOMe [*F*_(5,42)_ = 26.76, *p* < 0.0001], 25B-NBOMe [*F*_(5,42)_ = 15.50, *p* < 0.0001] (Figure 8A), and LSD [*F*_(5,42)_ = 16.76, *p* < 0.0001] (Figure 8C). The 2C compounds (0.001–10 mg/kg, i.p.) depressed the time spent in the C zone of mice at higher doses (Figure 8B). One-way ANOVA showed a significant effect of treatment for 2C-H [*F*_(5_,_42)_ = 4.594, *p* = 0.0020], 2C-I [*F*_(5,42)_ = 5.825, *p* = 0.0004], and 2C-B [*F*_(5,42)_ = 4.785, *p* = 0.0015].

##### Correlation Between Sensory Dysperception and Distance Traveled

**FIGURE 9:**
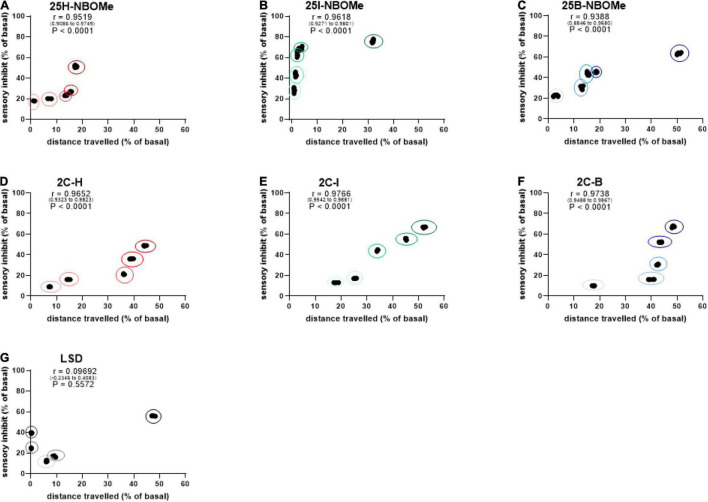
Correlation between sensory dysperception and total distance traveled by mice following administration of 25H-NBOMe (0.001–10 mg/kg i.p.) **(A)**, 25I-NBOMe (0.001–10 mg/kg i.p.) **(B)**, 25B-NBOMe (0.001–10 mg/kg i.p.) **(C)**, 2C-H. (0001–10 mg/kg i.p.) **(D)**, 2C-I (0.001–10 mg/kg i.p.) **(E)**, 2C-B (0.001–10 mg/kg i.p.) **(F)**, and LSD (0.001–10 mg/kg i.p.) **(G)** observed after 1 h. Data are expressed as mean ± SEM (*n* = 8/group). Statistical analysis was performed by XY correlation, which revealed a correlation between the two different effects of treatment for *p* < 0.0001 (2C-H, 2C-I, 2C-B, 25H-NBOMe, 25I-NBOMe and 25B-NBOMe) and *p* = 0.5572 (LSD).

To investigate whether the inhibition of distance traveled by mice within the first 30 min after administration of -NBOMe, 2C compounds, and LSD was directly proportional to sensory inhibition, we evaluated a possible correlation between these parameters. The results showed that there was a dose-dependent correlation in the first 30 min after administration of the -NBOMe and 2C compounds, but not for LSD. The administration of the -NBOMe series showed different results. XY correlation test revealed a correlation “r” [*r*_(0_._9519–0_._9749)_ = 0.9519, *p* < 0.0001] between the two different effects of treatment for 25H-NBOMe (0.001–10 mg/kg i.p.; Figure 9A). The 25H-NBOMe appeared to have a correlation profile similar to that of 2C-H (Figure 9D). However, the results obtained for 25I-NBOMe (0.001–10 mg/kg i.p.; Figure 9B) showed a higher sensory inhibition profile than the other substances tested. XY correlation test revealed a correlation “r” between the two different effects of treatment [*r*_(0_._9271–0_._9801)_ = 0.9618, *p* < 0.0001]. The administration of low dosages (0.001–1 mg/kg) showed a crescent and powerful sensory inhibition but minimal motor inhibition, while at a dose of 10 mg/kg, the motor inhibition was more severe. Differently, the XY correlation test for 25B-NBOMe (0.001–10 mg/kg i.p.; Figure 9C) revealed a correlation “r” between the two different effects of treatment [*r*_(0_._8846–0_._9680)_ = 0.9388, *p* < 0.0001]. The 25B-NBOMe clearly increased sensory inhibition proportionately to the distance traveled by mice at low dosages (0.001–1 mg/kg), while at the highest dose tested (10 mg/kg) there was a more severe motor inhibition. The 2C compounds clearly showed a different effect of correlation. The XY correlation test for 2C-H (0.001–10 mg/kg i.p.; Figure 9D) revealed a correlation “r” between the two different treatments [*r*_(0_._9652–0_._9823)_ = 0.9652, *p* < 0.0001], showed a dose-dependent sensory inhibition (that reaches the peak of approximately 48% at the highest dose tested), and the distance traveled by the mice decreased proportionately in a dose-dependent manner (from 7 to 44% approximately). The presence of halogen caused a different trend of profile. XY correlation test for 2C-I (0.001–10 mg/kg i.p.; Figure 9E) revealed a correlation “r” between the two different effects of treatment [*r*_(0_._9542–0_._9881)_ = 0.9766, *p* < 0.0001], which indicates the most evident correlation, with a proportional decrease in the sensory and motion capacities of the mouse. However, the XY correlation test for 2C-B (0.001–10 mg/kg i.p.; Figure 9F) revealed a correlation “r” between the two different effects of treatment [*r*_(0_._9488–0_._9867)_ = 0.9738, *p* < 0.0001]. The direct correlation between sensory and motor inhibition starts from the dose of 0.01 mg/kg. Finally, the XY correlation test for LSD (0.001–10 mg/kg i.p.; Figure 9G) revealed a correlation “r” between the two different effects of treatment [*r*_(–0_._2346 to 0_._4083)_ = 0.09692, *p* = 0.5572] and showed a correlation between sensory and motor inhibition, only at low doses (0.001–0.01 mg/kg) and at the highest dose of 10 mg/kg, but not for intermediate doses of 0.1 and 1 mg/kg.

### Startle/Prepulse Inhibition Studies

**FIGURE 10:**
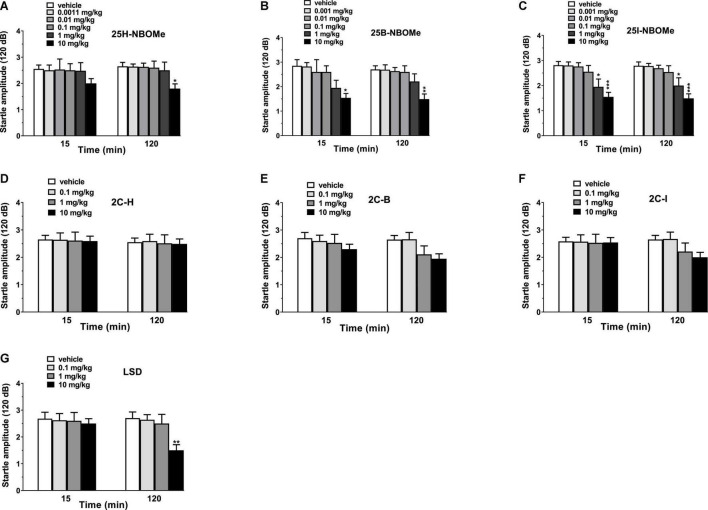
Effect of 25H-NBOMe (0.001–10 mg/kg i.p.) **(A)**, 25B-NBOMe (0.001–10 mg/kg i.p.) **(B)**, 25I-NBOMe (0.001–10 mg/kg i.p.) **(C)**, 2C-H (0.1–10 mg/kg i.p.) **(D)**, 2C-I (0.1–10 mg/kg i.p.) **(E)**, 2C-B (0.1–10 mg/kg i.p.) **(F)**, and LSD (0.1–10 mg/kg i.p.) **(G)** on startle amplitude in mice. Startle amplitude was expressed in absolute values (in dB) and the values represent the mean ± SEM of 10 animals for each treatment. The statistical analysis was performed with a one-way ANOVA followed by Bonferroni’s test for multiple comparisons. *p < 0.05, **p < 0.01 and ***p < 0.001 vs. vehicle.

Vehicle injection did not change the startle/PPI response in mice (Figures 10A–G). Administration of -NBOMe series (0.001–10 mg/kg, i.p.) and LSD (0.1–10 mg/kg, i.p.) caused an impairment of acoustic startle reflex (Figures 10A–C,G), while 2C compounds (0.1–10 mg/kg, i.p.) did not affect the startle amplitude in mice (Figures 10D–F). In fact, -NBOMe series administration showed a decrease in the startle response of mice at higher doses (1–10 mg/kg; Figures 10A–C). One-way ANOVA detected a significant effect of treatment for 25H-NBOMe (Figure 10A) at 120 min [*F*_(5,54)_ = 2.733, *p* = 0.0284]; for 25B-NBOMe (Figure 10B) at 15 min [*F*_(5,54)_ = 3.177, *p* = 0.0138] and at 120 min [*F*_(5,54)_ = 4.714, *p* = 0.0012]; and for 25I-NBOMe at 15 min [*F*_(5,54)_ = 6.597, *p* < 0.0001] and at 120 min [*F*_(5,54)_ = 6.993, *p* < 0.0001] (Figure 10C). LSD decreased the startle amplitude in mice only at highest dose (10 mg/kg; Figure 10G). One-way ANOVA detected a significant effect of treatment [*F*_(3,36)_ = 5.097, *p* = 0.0048] at 120 min.

**FIGURE 11:**
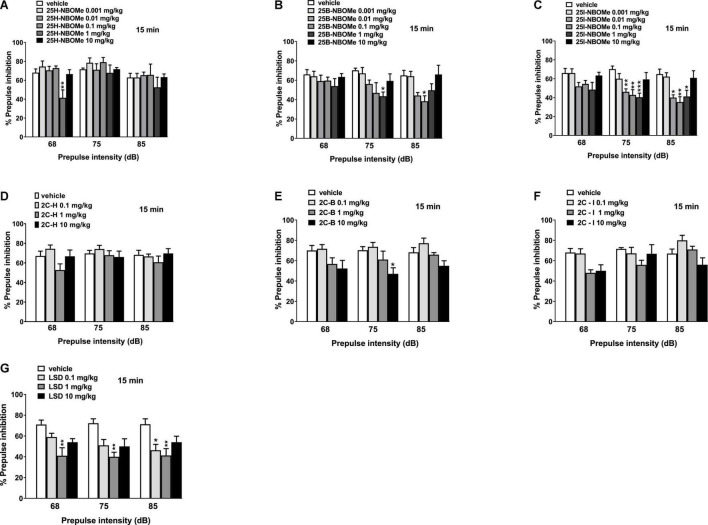
Effect of 25H-NBOMe (0.001–10 mg/kg i.p.) **(A)**, 25B-NBOMe (0.001–10 mg/kg i.p.) **(B)**, 25I-NBOMe (0.001–10 mg/kg i.p.) **(C)**, 2C-H (0.1–10 mg/kg i.p.) **(D)**, 2C-I (0.1–10 mg/kg i.p.) **(E)**, 2C-B (0.1–10 mg/kg i.p.) **(F)**, and LSD (0.1–10 mg/kg i.p.) **(G)** on prepulse inhibition (PPI) in mice. Effects on PPI are shown for the three prepulse intensities (68, 75, and 85 dB) at 15 min after treatment. PPI was expressed as the percentage decrease in the amplitude of the startle reactivity caused by presentation of the prepulse (% PPI; see section “Material and Methods”) and values represent mean ± SEM of 10 animals for each treatment. The statistical analysis was performed with a one-way ANOVA followed by Bonferroni’s test for multiple comparisons. *p < 0.05, **p < 0.01 and ***p < 0.001 vs. vehicle.

Notably, 25H-NBOMe increased the impairment of prepulse intensity in mice only at a dose of 1 mg/kg at 68 dB 15 min after injection (Figure 11A; significant effect of treatment [*F*_(5,48)_ = 5.441, *p* = 0.0005]. Systemic administration of 25B-NBOMe and 25I-NBOMe caused a decrease in prepulse intensity at lower doses (0.01–1 mg/kg), both at 75 dB and 85 dB (Figures 11B,C) 15 min after injection. There was a significant effect of treatment at 75 dB [*F*_(5,48)_ = 2.818, *p* = 0.0261] and 85 dB [*F*_(5,48)_ = 3.806, *p* = 0.0055] for 1 mg/kg of 25B-NBOMe. There was a significant effect of treatment of 25I-NBOMe (0.01–1 mg/kg) at 75 dB [*F*_(5,48)_ = 5.423, *p* = 0.0005] and at 85 dB [*F*_(5,48)_ = 5.780, *p* = 0.0003]. The 2C-H and 2C-I do not disrupt the sensorimotor gating significantly compared to the vehicle-treated group 15 min after administration (Figures 11D,F). In contrast, 2C-B tends to increase the impairment of prepulse intensity only at 75 dB at a dose of 10 mg/kg (Figure 11E). The effect was significant 15 min after the treatment [*F*_(3,32)_ = 4.185, *p* < 0.0131]. LSD increased the impairment of prepulse intensity in mice (Figure 11G). One-way ANOVA detected a significant effect of treatment at 15 min for different prepulse intensities: at 68 dB and a dose of 1 mg/kg [*F*_(3,32)_ = 6.016, *p* < 0.0023], at 75 dB and a dose of 1 mg/kg [*F*_(3,32)_ = 6.092, *p* < 0.0021], and at 85 dB and a dose of 0.1 mg/kg and 1 mg/kg [*F*_(3,32)_ = 5.093, *p* < 0.0054].

**FIGURE 12:**
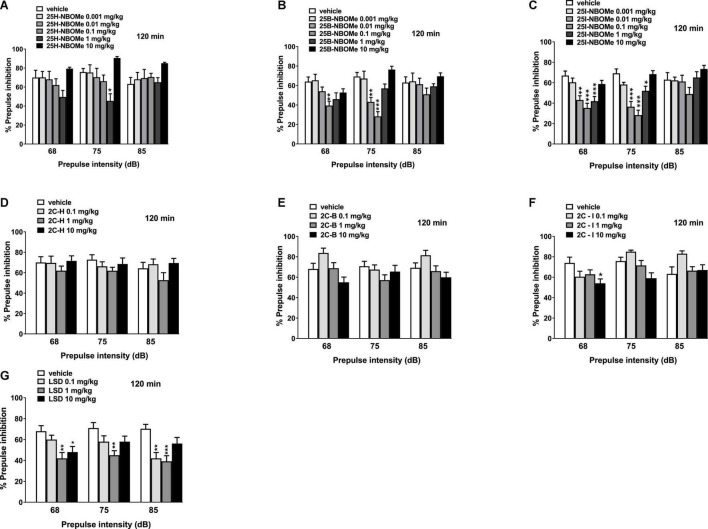
Effect of 25H-NBOMe (0.001–10 mg/kg i.p.) **(A)**, 25B-NBOMe (0.001–10 mg/kg i.p.) **(B)**, 25I-NBOMe (0.001–10 mg/kg i.p.) **(C)**, 2C-H (0.1–10 mg/kg i.p.) **(D)**, 2C-I (0.1–10 mg/kg i.p.) **(E)**, 2C-B (0.1–10 mg/kg i.p.) **(F)**, and LSD (0.1–10 mg/kg i.p.) **(G)** on prepulse inhibition (PPI) in mice. Effects on PPI are shown for the three prepulse intensities (68, 75, and 85 dB) 120 min after treatment. PPI was expressed as the percentage decrease in the amplitude of the startle reactivity caused by presentation of the prepulse (% PPI; see section “Material and Methods”) and values represent mean ± SEM of 10 animals for each treatment. The statistical analysis was performed with a one-way ANOVA followed by Bonferroni’s test for multiple comparisons. *p < 0.05, **p < 0.01 and ***p < 0.001 vs. vehicle.

The 25H-NBOMe increased the impairment of prepulse intensity in mice at only one dose (1 mg/kg) at 75 dB 120 min after injection (Figure 12A). One-way ANOVA detected a significant effect of treatment [*F*_(5,48)_ = 4.941, *p* = 0.0010]. After systemic administration of 25B-NBOMe (Figure 12B), one-way ANOVA detected a significant effect of treatment for 0.1 mg/kg at 68 dB [*F*_(5,48)_ = 3.893, *p* = 0.0048] and for 0.01 mg/kg and 0.1 mg/kg at 75 dB [*F*_(5,48)_ = 12.90, *p* < 0.0001], 120 min after injection. Lastly, 25I-NBOMe (0.001–10 mg/kg, i.p.) decreased the prepulse intensity in mice at lower doses (0.01–1 mg/kg) 120 min after injection (Figure 12C). There was a significant effect of treatment at 68 dB [*F*_(5,48)_ = 8.911, *p* < 0.0001] and 75 dB [*F*_(5,48)_ = 15.94, *p* < 0.0001]. The 2C-H and 2C-B do not disrupt the sensorimotor gating significantly compared with the vehicle-treated group 120 min after administration (Figures 12D,E). In contrast, 2C-I increased the impairment of prepulse intensity only at a high dose of 10 mg/kg at 68 dB (Figure 12E). One-way ANOVA detected a significant effect of treatment [*F*_(3,32)_ = 2.842, *p* < 0.0533] at 120 min. LSD decreased the prepulse intensity in mice at lower doses (0.1 mg/kg and 1 mg/kg) 120 min after injection (Figure 12G). One-way ANOVA detected a significant effect of treatment for different doses: at 68 dB for 1 mg/kg and 10 mg/kg [*F*_(3,32)_ = 5.158, *p* < 0.0051], at 75 dB only for a dose of 1 mg/kg [*F*_(3,32)_ = 4.237, *p* < 0.0125], and at 85 dB for low doses 0.1 mg/kg and 1 mg/kg [*F*_(3,32)_ = 7.355, *p* < 0.0007].

## Discussion

This study clearly presents a comparative analysis of the effects caused by new psychoactive substituted phenethylamines and LSD, showing a greater potency in the pharmaco-toxicological activity of -NBOMe. This is the first study in which the strong correlation between sensorimotor (visual and acoustic) inhibition and reaction time is highlighted. However, the inactivity of the substances on motor performance combined with their ability to significantly interrupt sensory gating (PPI), noticeably translates into a “trance-like” human state.

Our study clearly shows that the systemic administration of 25H-NBOMe and its halogenated derivatives (25I-NBOMe and 25B-NBOMe) have a time course, dose-dependence, and more potent effects than 2C compounds on mice. This results are in line with previous studies that examined the *in vivo* effects of -NBOMes and 2C compounds in mice (22, 23, 34). In particular, 25I-NBOMe and 25B-NBOMe heavily impaired sensorimotor (visual and acoustic), reaction time, and sensory gating (PPI) responses in CD-1 male mice. However, the halogenated derivatives of 25H-NBOMe appear to be mainly similar to LSD for both sensorimotor and sensory gating variation effects in mice. Frequently, halogenation represents a very common approach for NPS improvement (46, 47), and is especially used to provide more potent psychotropic effects for users. For example, studies have shown that para-halogenation of phenylethylamines improves their pharmacological effects (48).

### Major Behavioral Effects

Among the sensory effects observed after administration of 25H-NBOMe, 25I-NBOMe, 25B-NBOMe, 2C-H, 2C-I, 2C-B, and LSD, the most marked were the inhibition of visual responses (observed in both placing and object tests) and the acoustic response. The results obtained in the visual test clearly show that the halogenated compounds (25I-NBOMe and 25B-NBOMe) have a more pronounced dose-dependent response than that of 25H-NBOMe, especially in the object response test (Figures 2A–C). The same happened with the 2C compounds: 2C-H was among the least powerful compounds at all doses tested, and the object response test was effective only at the highest dosage (Figure 2D), while the placing test was ineffective (Figure 3A). In contrast, halogenated derivatives (2C-I and 2C-B) show a clear dose-dependent response inhibition profile (Figures 2E,F, 3E,F). In fact, our results are in line with previous studies (26). LSD seems to have a clearer profile of inhibition: the curves demonstrate a dose-dependent effect over time, especially in visual placing (Figure 3G), although the greatest inhibition effect is noted in the visual object test at the highest dose administered (Figure 2G). However, the two comparison graphs of maximum and average effect (Figures 2H,I, 3H,I) show how the dose-dependent response trend of the -NBOMe class and, in particular, its halogenated derivatives, is similar, if not at times stronger, to that of LSD. Nevertheless, the visual placing test showed an unusual response in which 25H-NBOMe induced more potent effect respect to the other compounds. In fact, the -NBOMe potency rank order is inverted only in this test. This is probably due to the different circuits involved in visual placing test. In particular, vestibular pathways together with other circuits (visual, tactile and motor one) play an important role in mice response (49). Thus, further studies to investigate this evidence should be undertaken.

Other important behavioral effects were detected through tests carried out to verify the acoustic functions of the animal (Figure 4). Contrary to LSD (Figure 4G), all phenethylamines tested are capable of inducing (some more and some less) a significant reduction in the animal’s response to sound stimuli. The halogenated compounds of -NBOMe and 2C compounds showed a delayed inhibitory effect (Figures 4B,C,E,F) that was once again more effective than that of the parent 25H-NBOMe (Figure 4A) and 2C-H (Figure 4D). This time, from the comparison graphs of the maximum and average effects of all tested compounds, it is clear that 25I-NBOMe has a higher inhibitory effect (Figures 4H,I). Results are in accordance with previous studies conducted with other hallucinogenic substances, such as DOI (50) and 25I-NBOMe (37) on rats, Dimethoxybromoamphetamine (DOB), and Paramethoxyamphetamine (PMA) on mice (38). Evidence accumulated over the past 5 decades has indicated that the behavioral effects of hallucinogenic compounds are mediated by interactions with serotonin receptors in the brain (10, 15, 32, 51, 52), resulting in exceptionally strong behavioral and psychoactive properties in animals and humans (12, 34, 53, 54). Despite the visible structural difference (Figure 1), the ability of all substances tested in this study to act by binding to the 5-HT_2A_ receptor is widely recognized by *in vitro* tests, and the -NBOMe compounds are ultrapotent and highly efficacious agonists (over 100 times higher) compared with the 2C compounds [Table 3; (11, 21, 55, 59, 60)]. The binding of LSD to the 5-HT_2A_ receptor is also known to be very strong (26, 58, 61). In addition, the LSD conformation of the diethylamide part is optimal for binding a portion of the receptor which has a highly complementary structure (62). Therefore, considering the anatomical localization of 5-HT_2A_ receptors in the brain of primates, which are distributed in different areas (63), we hypothesized that the compound induced visual hallucinations to alter not only the animal’s own visual abilities but those integrating the visual process (e.g., the perception of space and object and in the specific case of approaching the floor). In agreement with studies by Kometer et al., which reported that serotoninergic receptors of subtype 2A play a crucial role in the mechanism of action of hallucinogenic compounds and mediate effects, such as audio-visual synesthesia and significant alteration of visual perception (64–66), our results showed the visual and acoustic inhibition responses. In addition, previous studies in mice have shown different effects induced on visual and acoustic parameters by halogenated compounds (43, 67). Moreover, in the dorsal region of the nucleus accumbens, 5HT2 receptors are activated by increasing the electrical activity of neurons, thus leading to a final suppression of the auditory process (68).

**TABLE 3:** Different EC_50_ and Ki values of LSD, 2C compounds, and -NBOMe series on human and rat receptors.

Hallucinogen compounds	EC50		Ki	
	Human	Rats	Human	Rats
25H- NBOMe	5HT2A:0.49 ± 0.07 μM (26)	/	5-HT1A: 6.0 ± 0.7 μM 5HT2A: 16.4 ± 1.4 nM (48)	5HT2A: 1,19 nM (9, 55)
25I- NBOMe	5HT2A:0.44 nM (9, 55)	5HT2A:0.0813 nM (9, 55)	5HT2A:0.044 ± 0.006 nM (9, 55)	5HT2A:0.087 ± 0.01 nM (9, 55)
25B-NBOMe	5HT2A: 40 ± 10 nM (26)	/	5-HT1A:3.6 ± 0.3μM 5HT2A:0.5 nM (48)	/
2C-H	5HT2A:9.4 ± 0.5 μM (26)	/	5-HT1A: 70 ± 20 nM 5HT2A:1.6 ± 0.3 μM (48)	/
2C-I	5HT2A: 2.54 nM (9, 55); 60 ± 30 nM (26)	/	5-HT1A: 180 ± 10 nM (48) 5HT2A: 0.62 nM (9, 55); 3.5 ± 1 nM (26)	/
2C-B	5HT2A:80 ± 20 nM (26)	/	5-HT1A: 240 ± 40 nM 5HT2A:8.6 ± 3 nM (48)	5-HT1A:320 ± 40 nM 5-HT1B:25 ± 5 nM 5-HT1C:36 ± 3 nM 5HT2:1 nM (56)
LSD	5HT2A:0.26 ± 0.15μM 5HT2B:12 ± 0.35μM (26) 5HT2C: 27 ± 9.2 (57)	/	5HT1A:0.003 ± 0.0005 μM 5HT2A:0.0042 ± 0.0013 μM 5HT2C:0.015 ± 0.003 μM D1: 0.31 ± 0.1 μM D2: 0.025 ± 0.0004 μM (58)	5HT1A: 1.1nM 5HT2A: 2.5 nM 5HT2C: 10 nM D1:27.1 nM D4:5 nM (58)

### Reaction Time

Control mice rapidly react (0.21 s) to the fall on a plane; however, following the administration of phenethylamine compounds, the reaction time increases. In fact, remarkable increase in the reaction time of the animal during the first hour of administration of 25I-NBOMe and 25B-NBOMe (Figures 5B,C) indicates a clearly more powerful profile of effect mediated by these compounds than not only their parent 25H-NBOMe (Figure 5A), but the previous 2C compounds and LSD (Figures 5D–G). These results highlight the dispersive and hallucinogenic action of the phenethylamines tested, as confirmed by previous studies in mice (34). Graph 6 shows that sensory inhibition and reaction time have a directly proportional and dose-dependent trend. The results obtained in this test could be predictive of the potential “trance-like” behavior, which is typical of users who abuse hallucinogenic substances. These results are fundamental to understanding the possible real risks of these substances to public health, especially because it can clearly lead to fatal episodes of driving under the influence of drugs (DUID), as already reported by previous studies (37, 69). Driving is an incredibly difficult task that requires the rapid integration and processing of various information using visual, cognitive, and motor tools; this task becomes extremely risky when one of these capacities fails. When driving while under the influence of drugs, such as hallucinogens, this is what occurs. Rajotte et al. described a case of 25C-NBOMe-impaired driving, in which the patient displayed multiple cognitive deficits, including an altered mental state with evidence of disorientation, light-headedness, and lack of awareness (69). Undoubtedly, all the reported signs and symptoms can severely impair driving performance, leading to the alteration of speed and movement perception, signaling ability, and other driving-related skills.

### Total Distance Traveled and Time Spent in the C Zone

The evaluation of total distance traveled by the animals showed that the administration of 25H- and 25B-NBOMe (Figures 7A,C), and 2C compounds (Figures 7D–F) are ineffective. In contrast, 25I-NBOMe (Figure 7B) and LSD (Figure 7G) increased spontaneous locomotor activity at lower (0.001–1 mg/kg) and intermediate (0.1–1 mg/kg) dosages. While both 25I-NBOMe and LSD inhibited mice responses at the highest dose tested (10 mg/kg), that resulted in a sharp decrease in spontaneous locomotor activity (Figures 7B,G). These results suggest that there is no direct dopaminergic effect or dopamine (DA) release mediated by 25H- and 25B-NBOMe, and 2C compounds. Our results are in agreement with studies on drugs that promote serotonergic transmission as 3,4-methylenedioxymethamphetamine [MDMA; (42)] or that act on the serotonergic receptor as 25I-NBOMe (37) and 2C-B (70) that induce a dispersive state that alters the visual and acoustic responses in rodents without affecting motor performance, especially at doses lower than 1 mg/kg. In contrast, some studies have shown opposite results, whereby doses of 25C- and 25I-NBOMe dose-dependent reduced locomotor activity in mice and rats (41, 71). Therefore, LSD also interacts with several 5-HT (e.g., 5-HT_1A_, 5-HT_2C_, 5-HT_5_, and 5-HT_7_) and DA (D_2l*ike*_) receptors [Table 3; (58); Rickli). The same applies to 25I-NBOMe that displays micromolar affinity for adrenergic (α1 and α2) and dopaminergic receptors (D1, D2, and D3; (4, 26)]. Previous studies have also investigated the attenuated stimulant effect of LSD in the exploratory activity of mice lacking the 5HT_5A_ receptor, suggesting that this receptor subtype may be involved in the psychostimulant effect of LSD (72, 73). Therefore, a study by Herian et al. confirmed that 25I-NBOMe may have effects on short-term memory, locomotor function, and anxiety (71). In addition, studies on locomotion and exploratory activity in rats showed an increase in response following the administration of DOI similar to that induced by LSD, despite influencing more different receptors; consequently the mechanisms of action of DOI and LSD may not be identical (74–76). Our results are in agreement with previous studies that confirm the different effects of similar molecules on rodents: DOI stimulates locomotion (77), while the DOB slightly inhibits it (38).

### Prepulse Inhibition

The role of 5-HT_2A_ receptor on sensorimotor gating modulation in mice has been recognized for years (78). Therefore, the pro-psychedelic effects of -NBOMe, 2C compounds, and LSD is also tested through the PPI. The present study demonstrates that all hallucinogenic compounds tested reduce the PPI in male mice, as reported by several studies with phenylalkylamine and other classical hallucinogens in rodents (20). Our study is in accordance with previous studies that demonstrated the disruption of PPI in male and female rats after administration of LSD (36) and 25I-NBOMe (37). The same has also been certified in mice following the administration of hallucinogenic substances, such as DOB and PMA (38). In fact, in our study, halogenated derivatives of the -NBOMe class (Figures 10B,C) appear to inhibit startle reflex from a dosage of 1 mg/kg either at 15 or 120 min after administration. In contrast, 2C compounds (Figures 10D–F) appear to be ineffective on the variation of the animal’s startle reflex, while LSD (Figure 10G) and 25H-NBOMe (Figure 10A) appear to have an effect only at 10 mg/kg in mice 120 min after administration.

The introduction of Prepulse considerably alters the response of mice: -NBOMe series show a clear psychotropic effect greater at low doses of the two halogenated compounds (Figures 11B,C, 12B,C) than the parent 25H-NBOMe (Figures 11A, 12A). The same applies to LSD (Figures 11G, 12G). However, the Prepulse slightly modified the response of mice after 2C compounds administration: 2C-B (Figure 11E) at 15 min after administration of 10 mg/kg, only at 75 dB seems to inhibit the response of mice; the same happens for 2C-I (Figure 12F) at 120 min.

This could be the reason why in literature there are few review papers related to overdose from LSD, despite several cases of suicide and homicide (79). In contrast, there are several reported cases of certified acute serotoninergic syndrome related to clinical toxicity and deaths due to the intake of newly synthesized hallucinogenic substances (80–84).

## Conclusion

The present investigation was conducted to examine the more potent pharmaco-toxicological properties of three -NBOMe series (25H-, 25I-, and 25B-NBOMe) with respect to their 2C compounds analogs and LSD. In fact, this study showed the marked dispersive state of -NBOMe series and highlighted the strong direct correlation between sensory inhibition (alter the visual and acoustic responses) and the reaction time that was capable of inducing mice, without affecting motor performance. The absence of motor incoordination in the presence of a corresponding sensorimotor deficit, with the aggravating factor that the substances studied are able to significantly interrupt the sensorimotor gating (PPI) of mice, is a clearly translational response of the potential “trance-like” behavior typical of users who abuse hallucinogenic substances. Therefore, the -NBOMe series is an important issue for public health and safety because sensorimotor impairment may clearly lead to fatal accidents due to users DUID.

## Data Availability Statement

The original contributions presented in the study are included in the article/Supplementary Material, further inquiries can be directed to the corresponding author/s.

## Ethics Statement

The animal study was reviewed and approved by the Italian Ministry of Health (license no. 335/2016-PR) and by the Animal Welfare Body of the University of Ferrara.

## Author Contributions

MM and MT contributed to the conception and design of the study. RA, SB, MT, GC, and BM performed *in vivo* experiments. MM, MT, and KG wrote the manuscript. RA, SB, MM, MT, TB, FB, GC, GS, FD-G, and BM edited sections of the manuscript. MT, MM, SB, RA, GC, and BM performed statistical analysis. All authors contributed to the manuscript revision, and read and approved the submitted version.

## Conflict of Interest

The authors declare that the research was conducted in the absence of any commercial or financial relationships that could be construed as a potential conflict of interest.

## Publisher’s Note

All claims expressed in this article are solely those of the authors and do not necessarily represent those of their affiliated organizations, or those of the publisher, the editors and the reviewers. Any product that may be evaluated in this article, or claim that may be made by its manufacturer, is not guaranteed or endorsed by the publisher.

## Abbreviations

LSD: Lysergic acid diethylamide
2C-H 2: 5-dimethoxyphenethylamine
2C-I 2: 5-dimethoxy-4-iodophenethylamine
2C-B 2: 5-dimethoxy-4-bromophenethylamine
25H-NBOMe 2: 5-dimethoxy-N-[(2-methoxyphenyl) methyl]-benzeneethanamine
25I-NBOMe 4-iodio-2: 5-dimethoxy-N-[(2-methoxyphenyl) methyl]-benzeneethanamine
25B-NBOMe 4-bromo-2: 5-dimethoxy-N-[(2-methoxyphenyl) methyl]-benzeneethanamine.
