# Supplementary material for: Effect of -NBOMe Compounds on Sensorimotor, Motor, and Prepulse Inhibition Responses in Mice in Comparison With the 2C Analogs and Lysergic Acid Diethylamide: From Preclinical Evidence to Forensic Implication in Driving Under the Influence of Drugs
Source: Front Psychiatry. 2022 Apr 21;13:875722. doi: 10.3389/fpsyt.2022.875722 (PMC9069068; doi:10.3389/fpsyt.2022.875722)
Supplement: Supplementary file 1 [file Data_Sheet_1.pdf]

Supplementary materials

Evaluation of the visual response

Visual response was verified by two behavioral tests which evaluated the ability of the animal to capture visual information when the animal is either stationary (the visual object response) or moving (the visual placing response).

Visual object response test evaluates the mouse's ability to see different objects approaching first from the front (front view) and then from the side (side view), thus causing the animal to react differently: move or turn the head, bring the forelimbs to the "defense" position or retreat it. For the frontal visual response, a white horizontal bar was moved frontally to the mouse head. For the lateral visual response, a small dentist's mirror was moved into the mouse's field of view in a horizontal arc, until the stimulus was between the mouse's eyes. The procedure was conducted bilaterally (Ossato et al., 2015; Marti et al, 2019; Bilel et al.,2020; Tirri et al., 2020). Both visual object testes are repeated three times and the score assigned was a value of 1 if there was a reflection in the mouse movement or 0 if not. The total value was calculated by adding the scores obtained in the frontal with that obtained in the lateral visual object response (overall score 9).

Visual Placing response test is performed using a tail suspension modified apparatus able to bring down the mouse towards the floor at a constant speed of 10 cm/sec (Ossato et al., 2015). Briefly, CD-1 mice were suspended 20 cm above the floor by an adhesive tape that was placed approximately 1 cm from the tip of the tail. The downward movement of the mouse is videotaped by a camera (B/W USB Camera day&night with varifocal lens; Ugo Basile, Italy) placed at the base of the tail suspension apparatus. Movies are analyzed off-line by a trained operator who does not know the drug treatments performed. The analysis frame by frame allows to evaluate the beginning of the reaction of the mouse while it is close to the floor. The test uses an electronic ruler that evaluates the perpendicular distance in millimeters between the floor and the first-mice movement reaction to extension of the front legs. The mice untreated control perceives the floor and it prepares to contact at a distance of about 26.8 ± 4.4 mm.

Evaluation of acoustic response

Acoustic response measures the reflex of the mouse in replay to an acoustic stimulus produced behind

the animal (Ossato et al., 2015). Four acoustic stimuli of different intensity and frequency were tested. A *snap of the fingers* (four snaps repeated in 1.5 sec), a *sharp click* (produced by a metal instrument; four clicks repeated in 1.5 sec), an *acute* (produced by an audiometer that reproduces a high-pitched sound at a frequency of around 5.0-5.1 kHz) and a *severe* (produced by an audiometer that reproduces a sound at a frequency of around 125-150 Hz) sound. Each test was repeated 3 times, giving a value of 1 if there was a response, 0 if not present, for a total score of 3 for each sounds. Acoustic test was performed at 0, 10, 30, 60, 120, 180, 240 and 300 min post injection. The acoustic total score was calculated by adding scores obtained in the four tests (overall score 12). The background noise (about 40±4 dB) and the sound from the instruments are measured with a digital sound level meter.

Evaluation of overall tactile response

The tactile response in the mouse was verified through vibrissae, pinna and corneal reflexes (Ossato et al., 2015). Data is expressed as the sum of the three above-mentioned parameters (overall score 12).

Vibrissae reflex was evaluated by touching vibrissae (right and left) with a thin hypodermic needle once for side giving a value of 1 if there was a reflex (turning of the head to the side of touch or vibrissae movement) or 0 if not present (overall score 2).

Pinna reflex was assessed by touching pavilions (left and right) with a thin hypodermic needle. First the interior pavilions and then the external. This test was repeated twice for side giving a value of 1 if there was a reflex and 0 if not present (overall score 4).

Corneal reflex was assessed gently touching the cornea of the mouse with a thin hypodermic needle and evaluating the response, assigning a value of 1 if the mouse moved only the head, 2 if it only closed the eyelid, 3 if it closed the lid and moved the head. The procedure was conducted bilaterally (overall score 6).

Evaluation of reaction time

This test is carried out in order to measure the animal's motor reactivity in the open field (Viaro et al., 2010). Mice are made accustomed to being in the centre of a square area (150 × 150 cm) for 5 minutes, then lifted from the tail for 3 cm above the surface and finally dropped. When the animal touches the floor, the latency time for the first movement of the front limb is recorded. As with previous tests, reaction time test is also taken by a camera (B/W USB Camera day&night with varifocal lens; Ugo Basile, Italy) and the films are analysed off-line by a qualified operator who does not know the pharmacological treatments performed. Frame-by-frame analysis allows to evaluate the start of the

mouse's reaction.

Evaluation of skeletal muscle strength

This test was used to evaluate the skeletal muscle strength of the mice (Viaro et al., 2010). The grip-strength apparatus (ZP-50N, IMADA) is comprised of a wire grid (5 × 5 cm) connected to an isometric force transducer (dynamometer). In the grip-strength test, mice were held by their tails and allowed to grasp the grid with their forepaws. The mice were then gently pulled backward by the tail until the grid was released. The average force exerted by each mouse before losing its grip was recorded. The mean of three measurements for each animal was calculated, and the mean average force was determined. The skeletal muscle strength is expressed in gram force (gf) and was recorded and processed using IMADA ZP-Recorder software.

Stimulated Motor activity assessment.

Alterations of stimulated motor activity induced by -NBOMe compounds, *2C analogs,* and *LSD* were measured using two tests in which the animal is forced to move the drag test and accelerod (Canazza et al., 2016; Bilel et al., 2020; De-Giorgio 2020). In the *drag* test the mouse was lifted by the tail, leaving the front paws on the table and dragged backward at a constant speed of about 20 cm/sec for a fixed distance (100 cm). The number of steps performed by each paw was recorded by two different observers. For each animal from five to seven measurements were collected. In the *accelerod* test, animals were placed for five-minute intervals on a rotating cylinder whose speed automatically and constantly increased (0-60 rotations/min). Time spent on the cylinder was then measured, placing a cutoff at 300 seconds.

Spontaneous locomotor activity

Spontaneous locomotor activity was measured by using the ANY-maze video-tracking system (application version 4.99g Beta, Stoelting Co. Europe, Dublin, Ireland). The mouse was placed in a square plastic cage (60 X 60 cm) located in a sound- and light-attenuated room and motor activity was monitored for 240 min. Four mice were monitored at the same time in each experiment. Parameters measured were: distance travelled (m), and time (sec) spent in central zone of arena (C1 zone). The distance travelled and the time in C1 zone were analyzed every 15 minutes for a maximum of 240 minutes. Subsequently, the registration of effect has been focused to study the time spent by the animals treated at the center of the cage. Therefore, the first hour following the administration has been considered. To avoid mice olfactory cues, cages were carefully cleaned with a dilute (5%) ethanol

solution and washed with water between animal trials. All experiments were performed between 9:00 AM to 1:00 PM.

Startle/Pre-Pulse inhibition analysis

Mice were tested for acoustic startle reactivity in startle chambers (Ugo Basile apparatus, Milan, Italy) consisting of a sound-attenuated, lighted and ventilated enclosure holding a transparent non-restrictive Perspex® cage (90 x 45 x 50 mm). A loudspeaker mounted laterally the holder produced all acoustic stimuli. Peak and amplitudes of the startle response were detected by a loadcell. At the onset of the startling stimulus, 300-ms readings were recorded and the wave amplitude evoked by the movement of the animal startle response was measured. Acoustic startle test sessions consisted of startle trials (pulse-alone) and prepulse trials (prepulse + pulse). The pulse-alone trial consisted of a 40-ms 120-dB pulse. Prepulse + pulse trials sequence consisted of a 20-ms acoustic prepulse, 80-ms delay, and then a 40-ms 120-dB startle pulse (100-ms onset–onset). There was an average of 15 seconds (range = from 9 to 21 seconds) between the trials. Each startle session began with a 10-min acclimation period with a 65-dB broadband white noise that was present continuously throughout the session. The test session contained 40 trials composed by pulse-alone and prepulse + pulse trials (with three different prepulses of 68-dB, 75-dB and 85-dB) presented in a pseudorandomized order. Animals were placed in the startle chambers 5 minutes after treatment. The entire PPI test lasted 20 minutes. The amount of prepulse inhibition (PPI) was expressed as the percentage decrease in the amplitude of the startle reactivity caused by the presentation of the prepulse (% PPI).

As previously reported (Marti et al., 2019; Miliano et al., 2019; Tirri et al., 2020) *LSD*, the -NBOMe compounds and *2C analogs* were administered intraperitoneally and startle/PPI responses were recorded 15 and 120 minutes (including the 10 minutes' acclimation period) after drug injections.

Figures

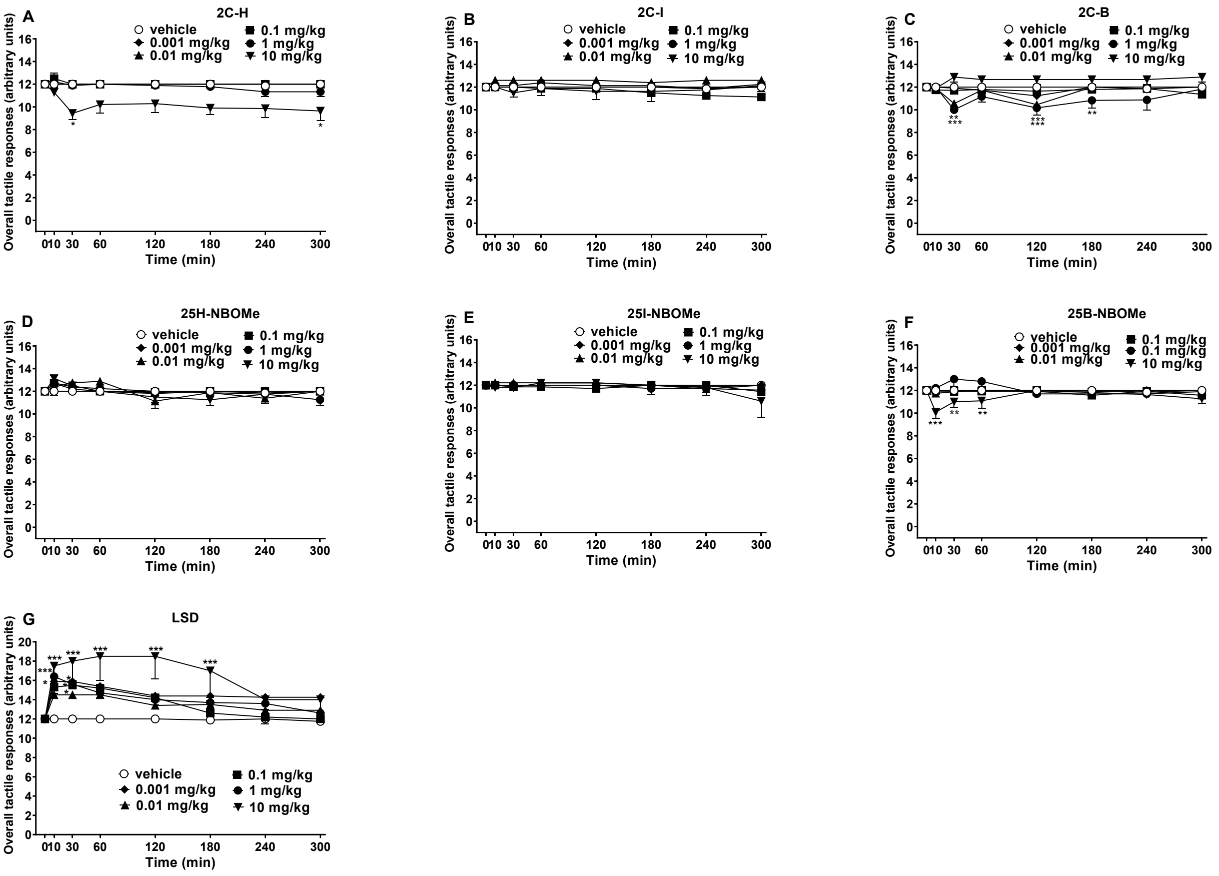

Figure 1: Effect of 2C-H (0.001–10 mg/kg i.p.; panel A), 2C-I (0.001–10 mg/kg i.p.; panel B), 2C-B (0.001–10 mg/kg i.p.; panel C), 25H-NBOMe (0.001–10 mg/kg i.p.; panel D), 25I-NBOMe (0.001–10 mg/kg i.p.; panel E), 25B-NBOMe (0.001–10 mg/kg i.p.; panel F), and LSD (0.001–10 mg/kg i.p.; panel G) on the overall tactile tests in mice observed in 5 hours. Data are expressed as mean ± SEM (n = 8/group). Statistical analysis was performed by two-way ANOVA followed by Bonferroni's test (panels A, B, C, D, E, F, and G) for multiple comparisons for the dose–response curve of each compound at different time points. *$p<0.05$, **$p<0.01$, ***$p<0.001$ *vs* vehicle.

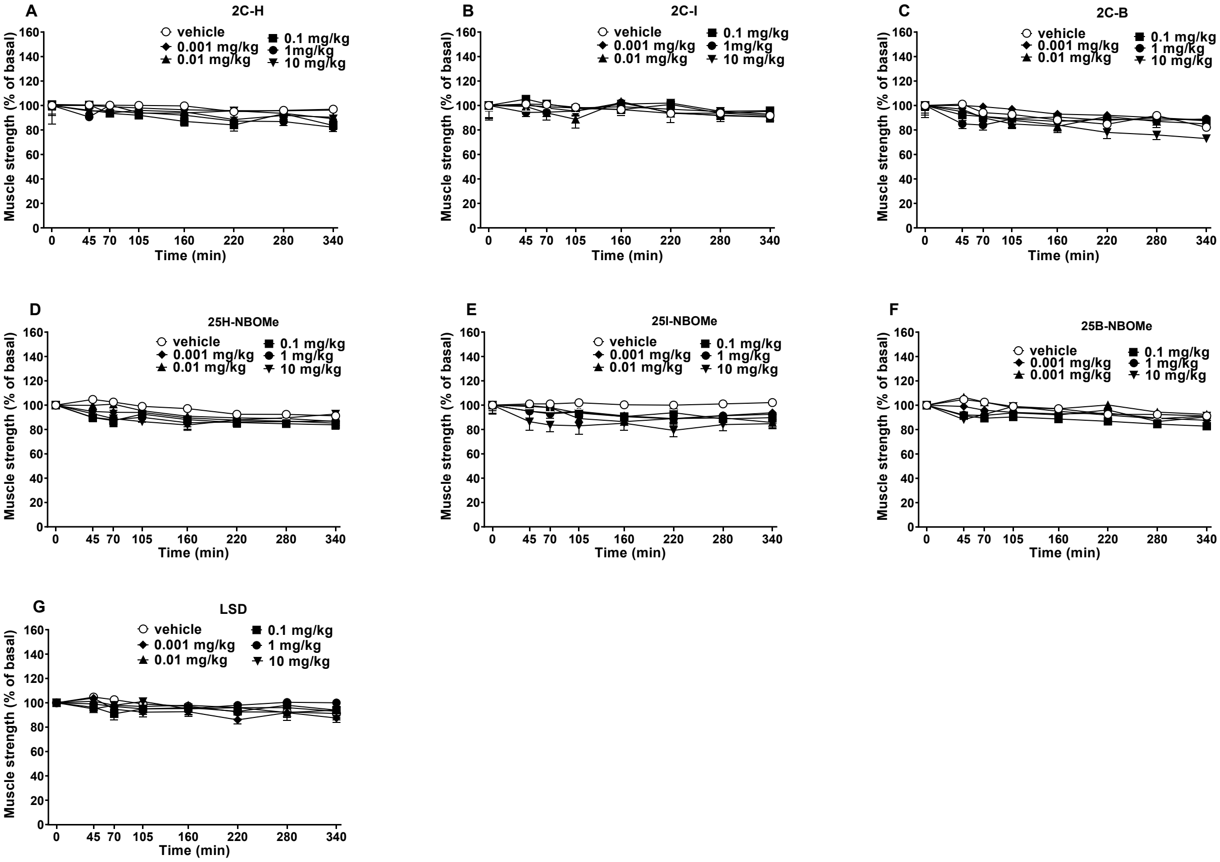

Figure 2: Effect of 2C-H (0.001–10 mg/kg i.p.; panel A), 2C-I (0.001–10 mg/kg i.p.; panel B), 2C-B (0.001–10 mg/kg i.p.; panel C), 25H-NBOMe (0.001–10 mg/kg i.p.; panel D), 25I-NBOMe (0.001–10 mg/kg i.p.; panel E), 25B-NBOMe (0.001–10 mg/kg i.p.; panel F), and LSD (0.001–10 mg/kg i.p.; panel G) on the muscle strength test in mice observed in 5 hours. Data are expressed as mean ± SEM (n = 8/group). Statistical analysis was performed by two-way ANOVA followed by Bonferroni's test (panels A, B, C, D, E, F, and G) for multiple comparisons for the dose–response curve of each compound at different time points.

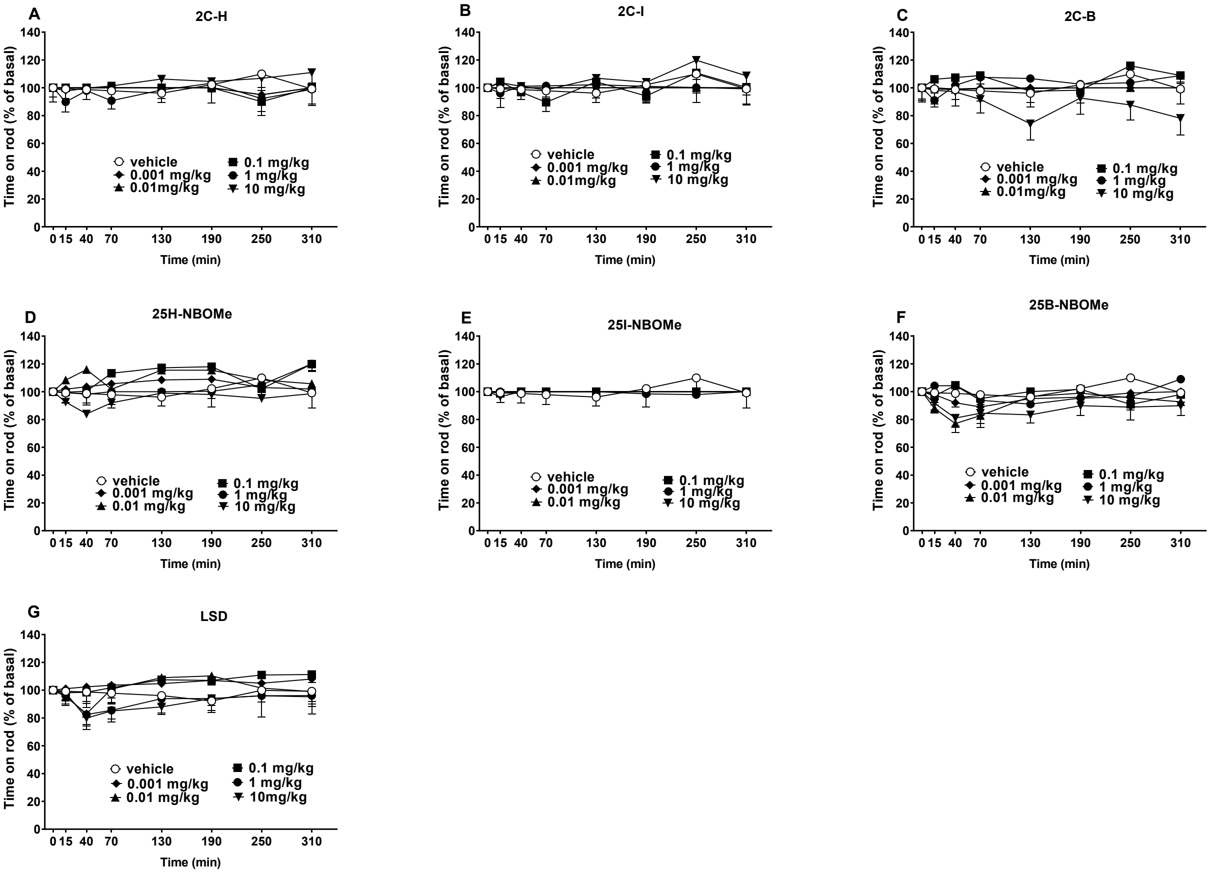

Figure 3: Effect of 2C-H (0.001–10 mg/kg i.p.; panel A), 2C-I (0.001–10 mg/kg i.p.; panel B), 2C-B (0.001–10 mg/kg i.p.; panel C), 25H-NBOMe (0.001–10 mg/kg i.p.; panel D), 25I-NBOMe (0.001–10 mg/kg i.p.; panel E), 25B-NBOMe (0.001–10 mg/kg i.p.; panel F), and LSD (0.001–10 mg/kg i.p.; panel G) on the accelerod test in mice observed in 5 hours. Data are expressed as mean ± SEM (n = 8/group). Statistical analysis was performed by two-way ANOVA followed by Bonferroni's test (panels A, B, C, D, E, F, and G) for multiple comparisons for the dose–response curve of each compound at different time points.

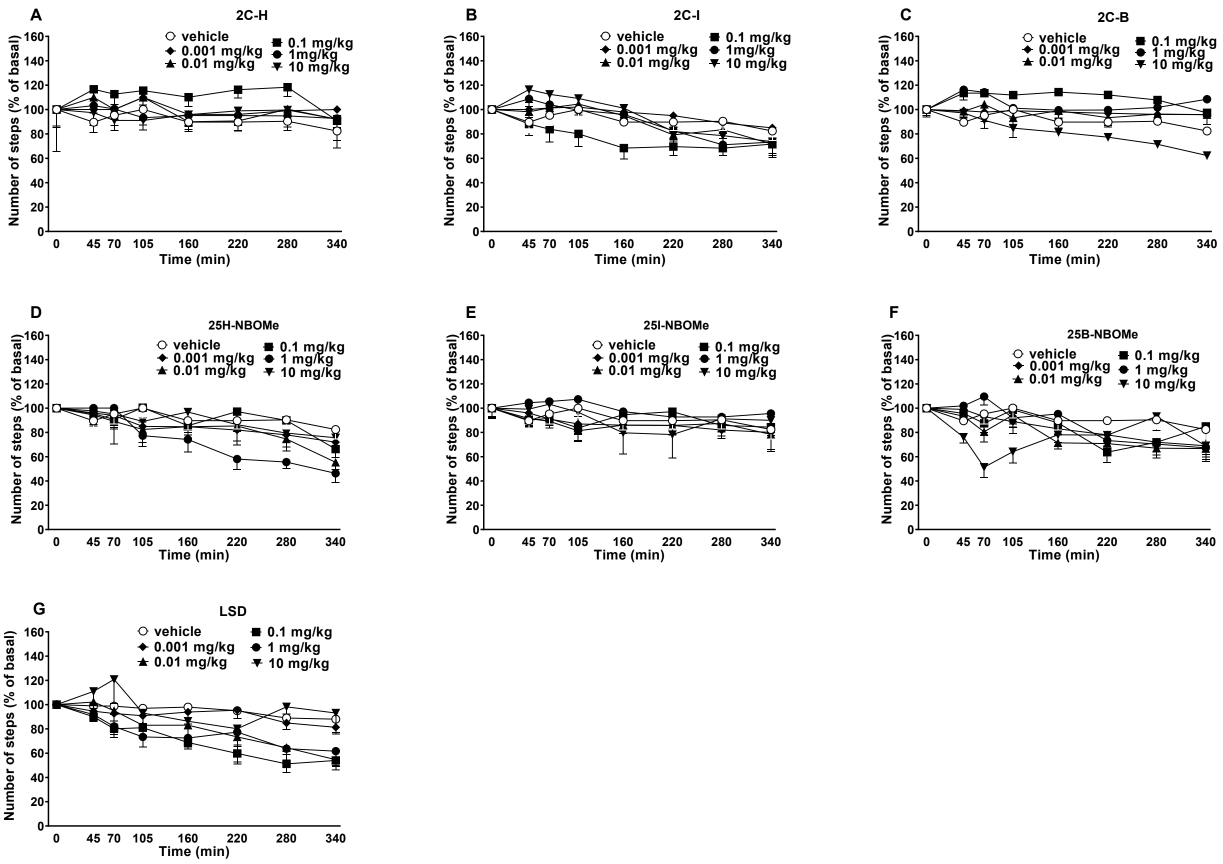

Figure 4: Effect of 2C-H (0.001–10 mg/kg i.p.; panel A), 2C-I (0.001–10 mg/kg i.p.; panel B), 2C-B (0.001–10 mg/kg i.p.; panel C), 25H-NBOMe (0.001–10 mg/kg i.p.; panel D), 25I-NBOMe (0.001–10 mg/kg i.p.; panel E), 25B-NBOMe (0.001–10 mg/kg i.p.; panel F), and LSD (0.001–10 mg/kg i.p.; panel G) on the drag test in mice observed in 5 hours. Data are expressed as mean ± SEM (n = 8/group). Statistical analysis was performed by two-way ANOVA followed by Bonferroni's test (panels A, B, C, D, E, F, and G) for multiple comparisons for the dose–response curve of each compound at different time points.
